# Supplementary material for: Synthesis of Novel Benzo[b][1,6]naphthyridine Derivatives and Investigation of Their Potential as Scaffolds of MAO Inhibitors
Source: Molecules. 2023 Feb 9;28(4):1662. doi: 10.3390/molecules28041662 (PMC9962805; doi:10.3390/molecules28041662)
Supplement: Supplementary file 1 [file molecules-28-01662-s001.zip › molecules-2213151-supplementary.pdf]

Supplementary Materials

Synthesis of Novel Benzo[*b*][1,6]naphthyridine Derivatives and Investigation of Their Potential as Scaffolds of MAO Inhibitors

Larisa N. Kulikova [1], Ghulam Reza Raesi [1], Daria D. Levickaya [1], Rosa Purgatorio [2], Gabriella La Spada [2], Marco Catto [2], Cosimo D. Altomare [2] and Leonid G. Voskressensky [1,*]

[1] Organic Chemistry Department, Peoples' Friendship University of Russia (RUDN University), Miklukho-Maklaya St. 6., 117198 Moscow, Russia

[2] Department of Pharmacy-Pharmaceutical Sciences, University of Bari Aldo Moro, Via E. Orabona 4, 70125 Bari, Italy

* Correspondence: lvoskressensky@sci.pfu.edu.ru; Tel.: +7-495-955-07-29

Table of Content

1. NMR Data of Derivatives (**3-10**)

2. A single crystal X-ray analysis of compound **5a**

Figure S1. The 1H NMR data of **3a**

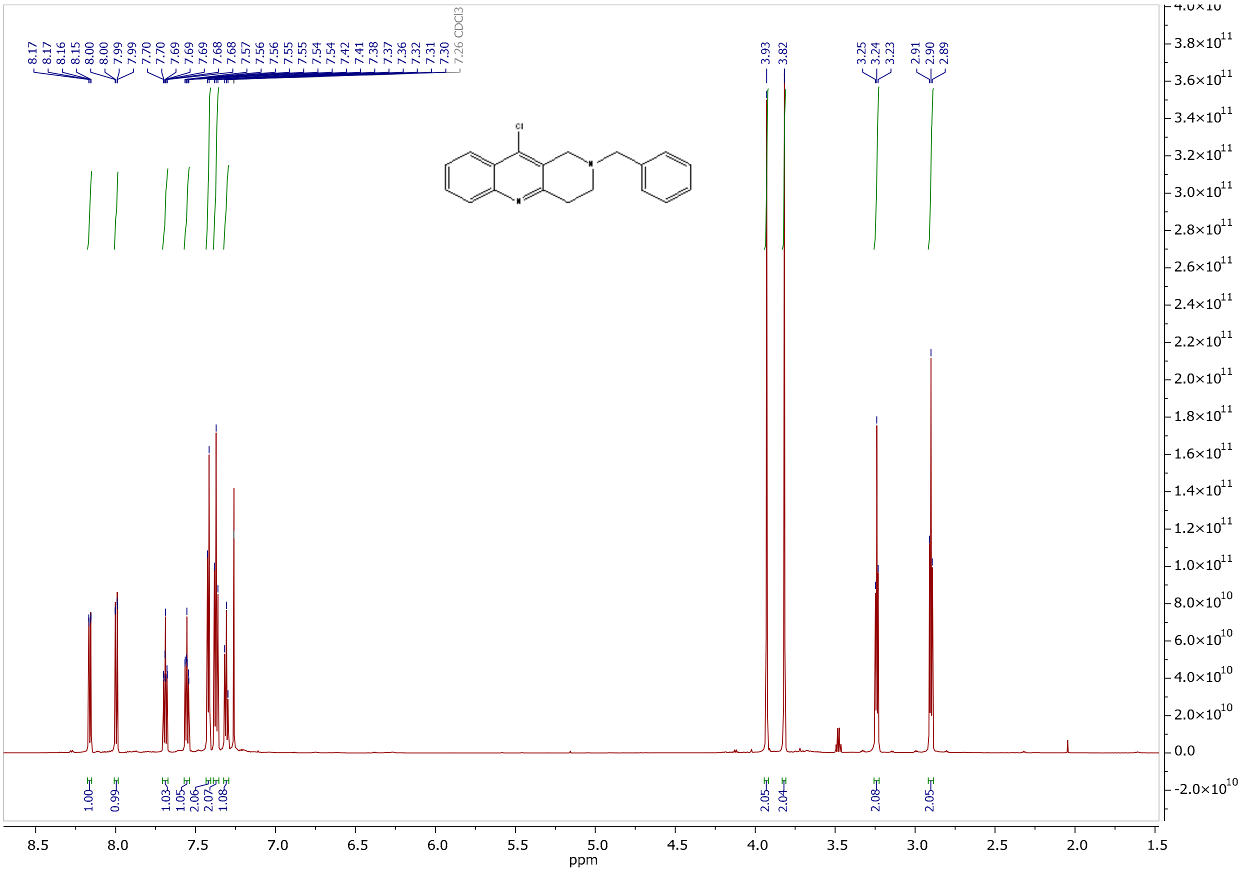

Figure S2. The 13C NMR data of **3a**

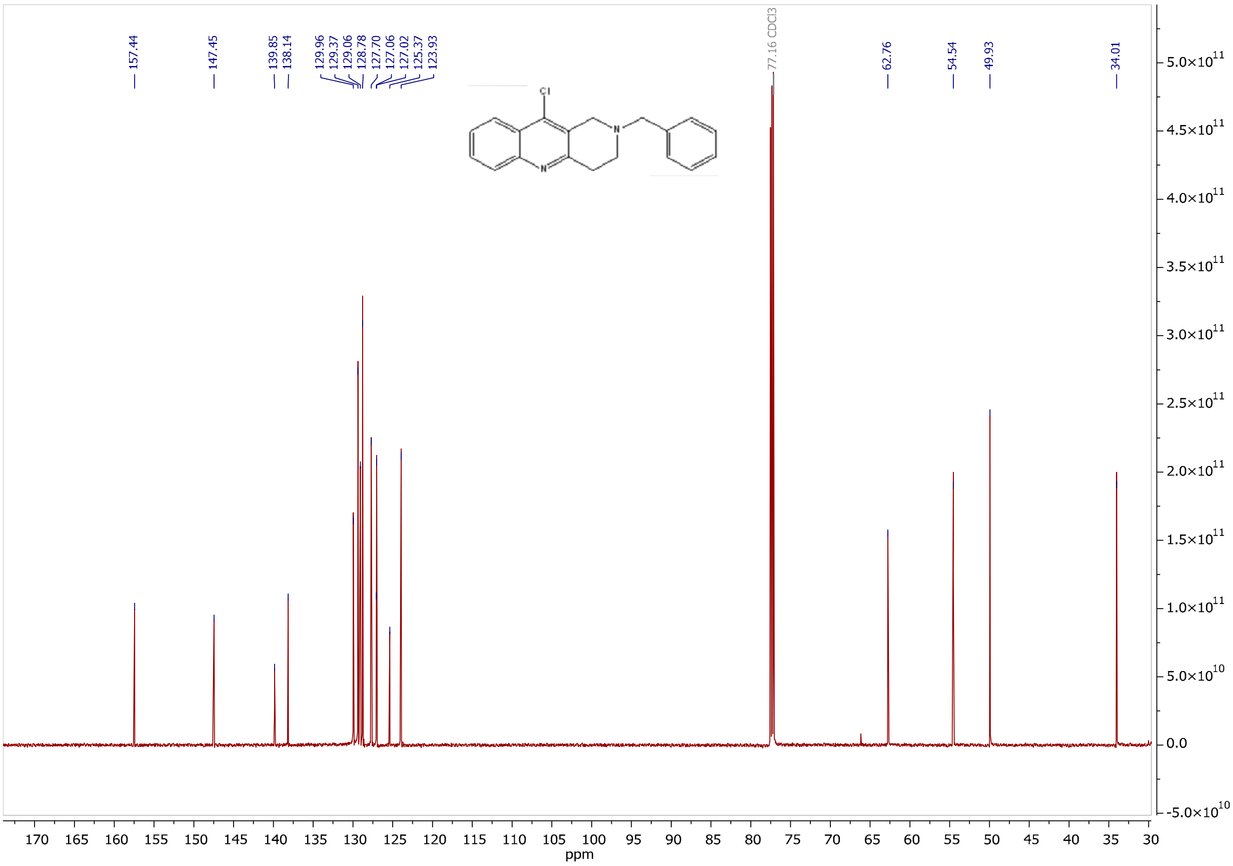

Figure S3. The 1H NMR data of **3b**

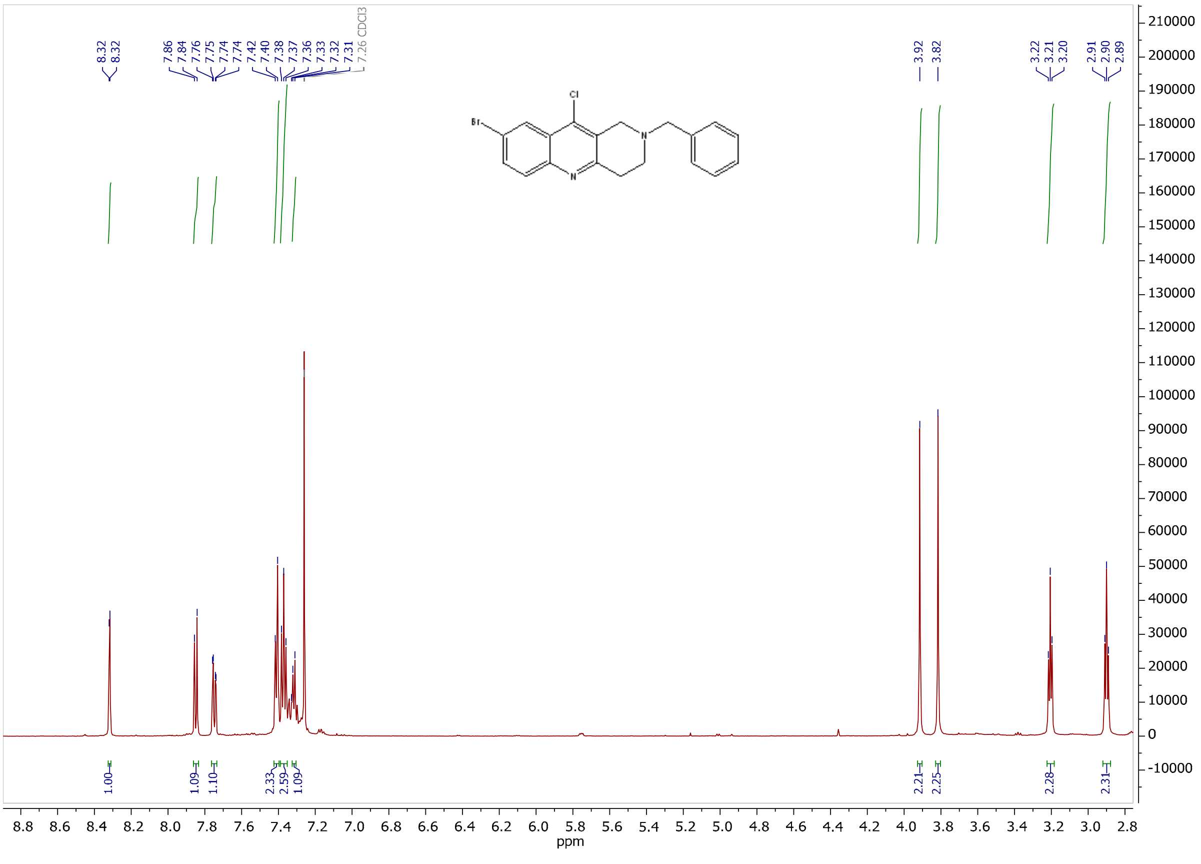

Figure S4. The 13C NMR data of **3b**

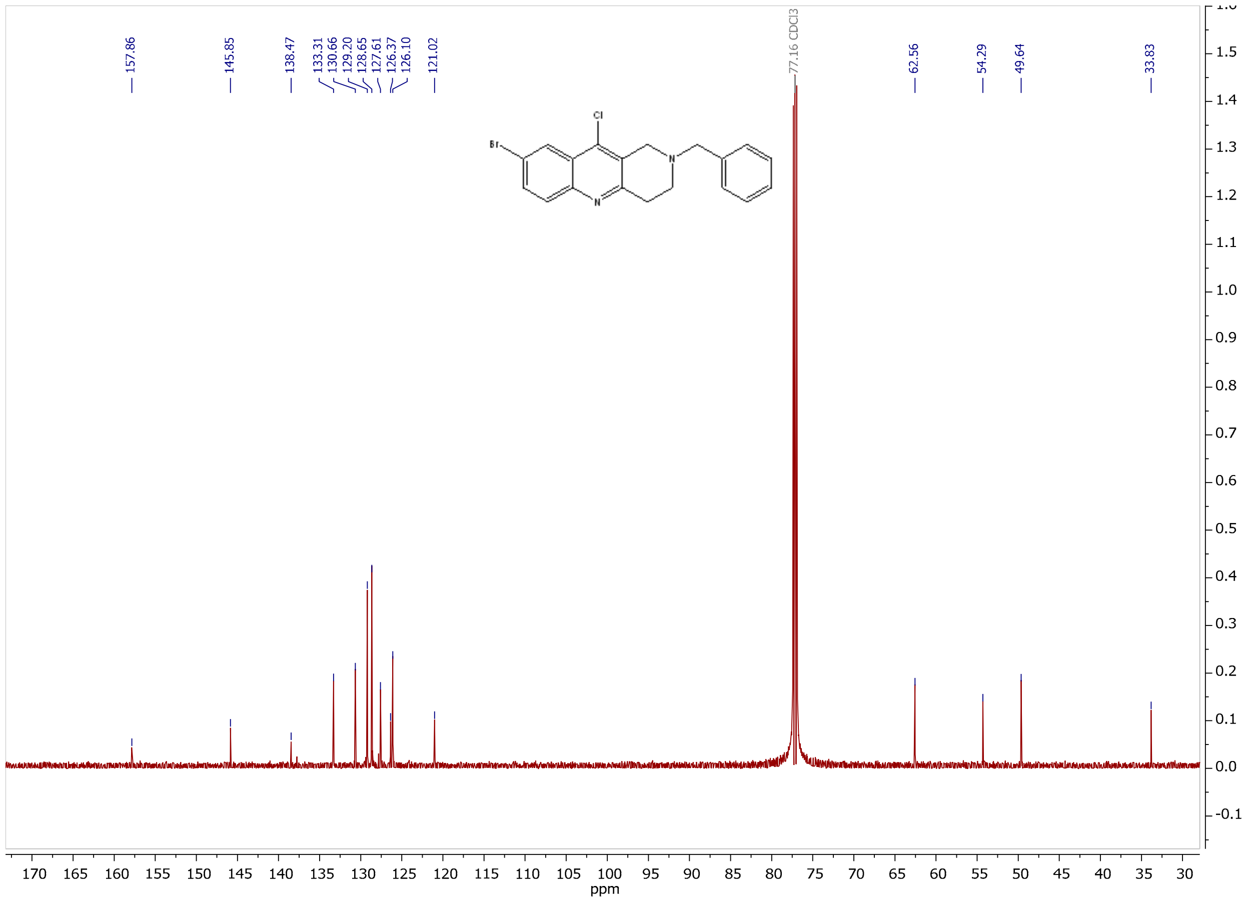

Figure S5. The 1H NMR data of **3c**

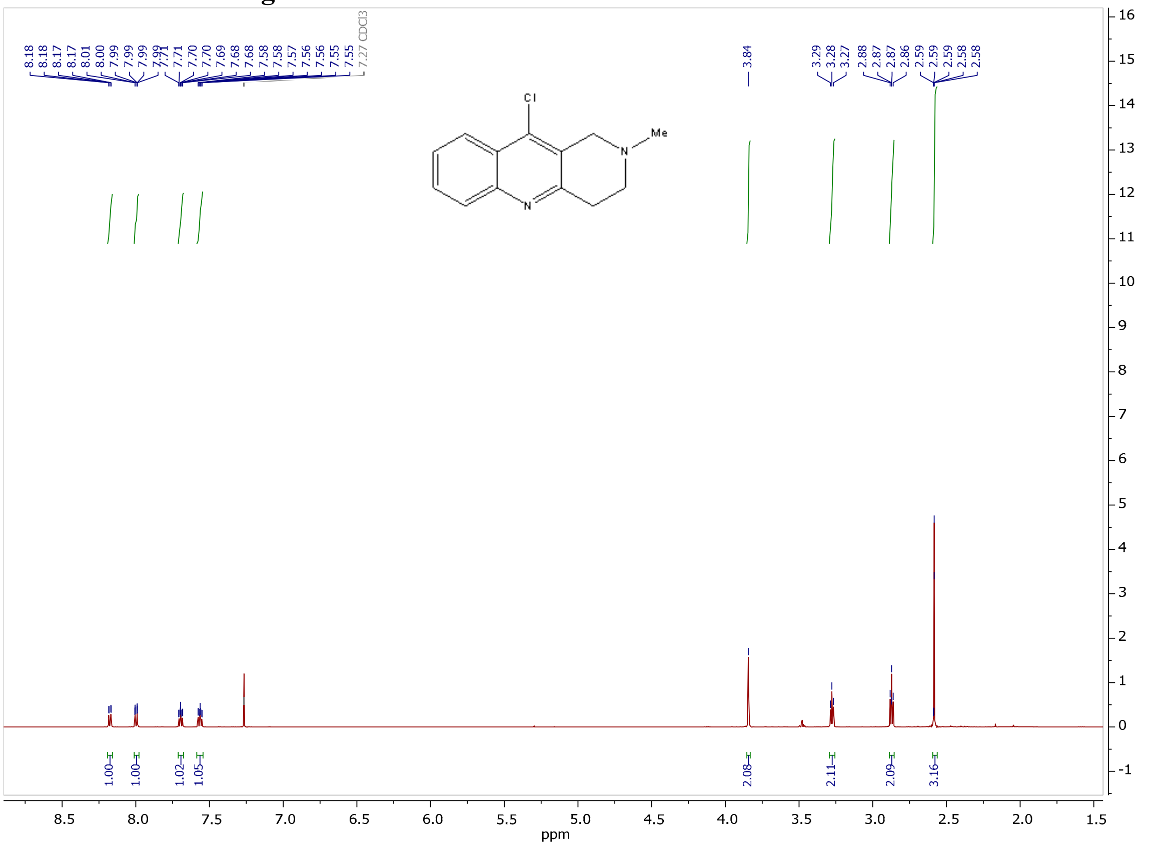

Figure S6. The 13C NMR data of **3c**

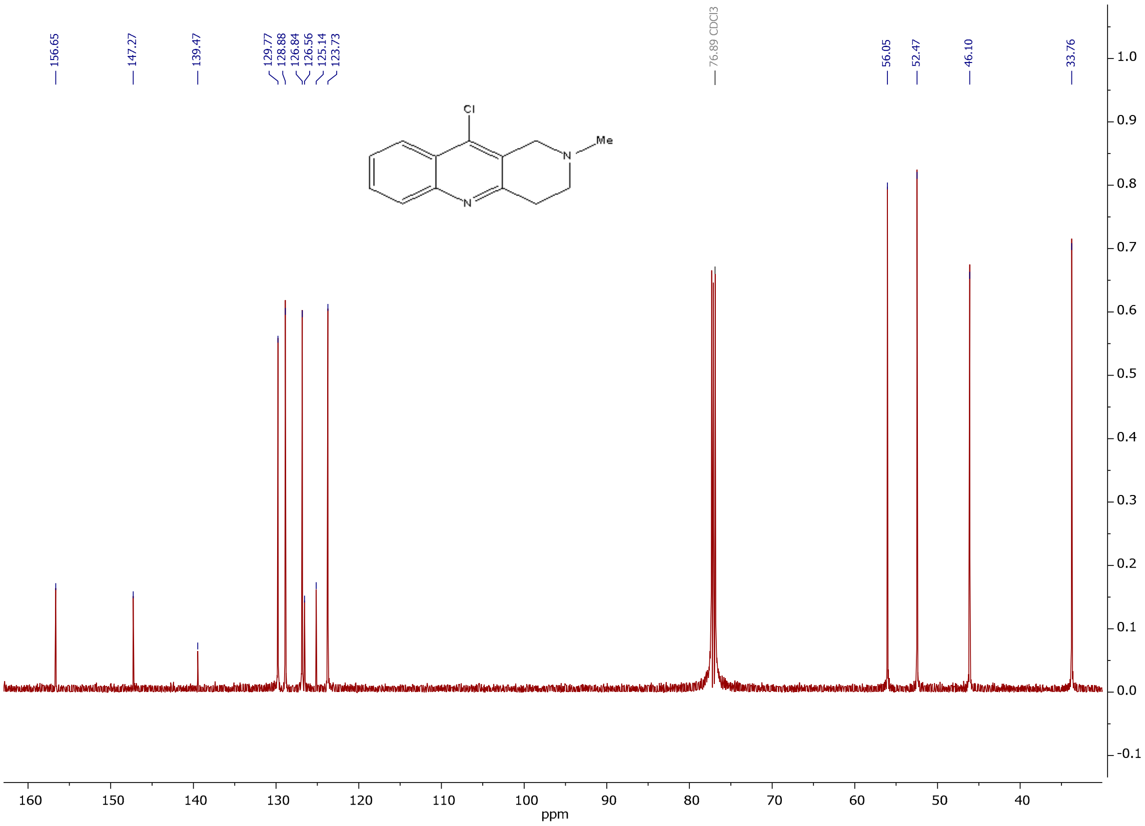

Figure S7. The 1H NMR data of **3d**

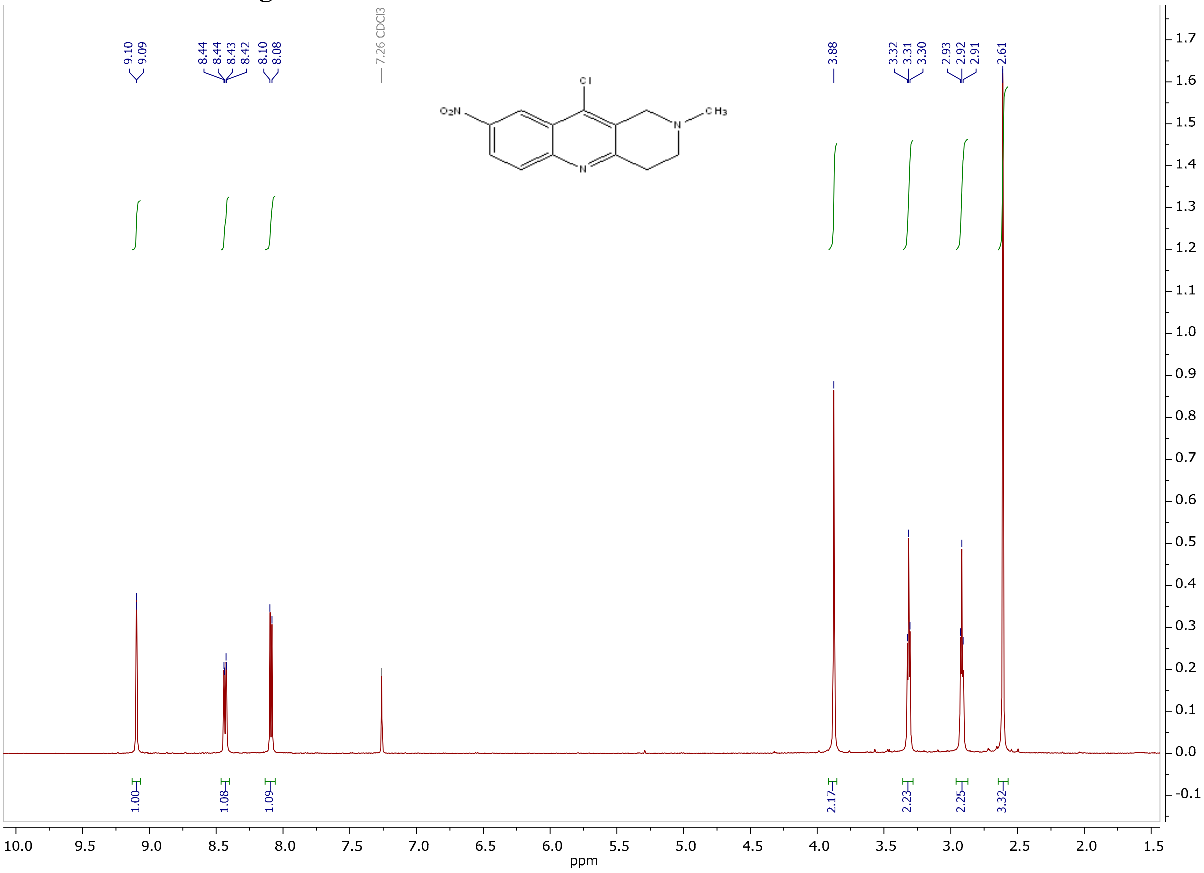

Figure S8. The 13C NMR data of **3d**

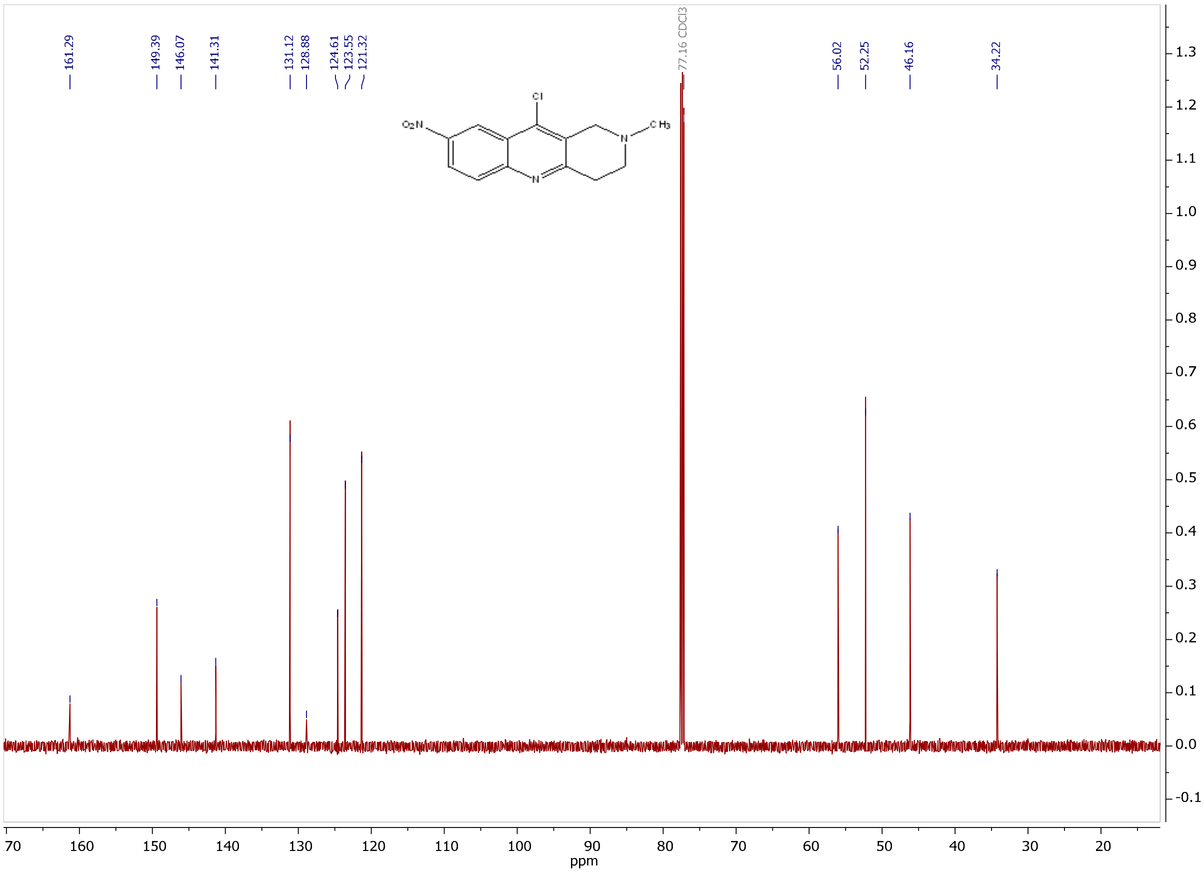

Figure S9. The 1H NMR data of **4a**

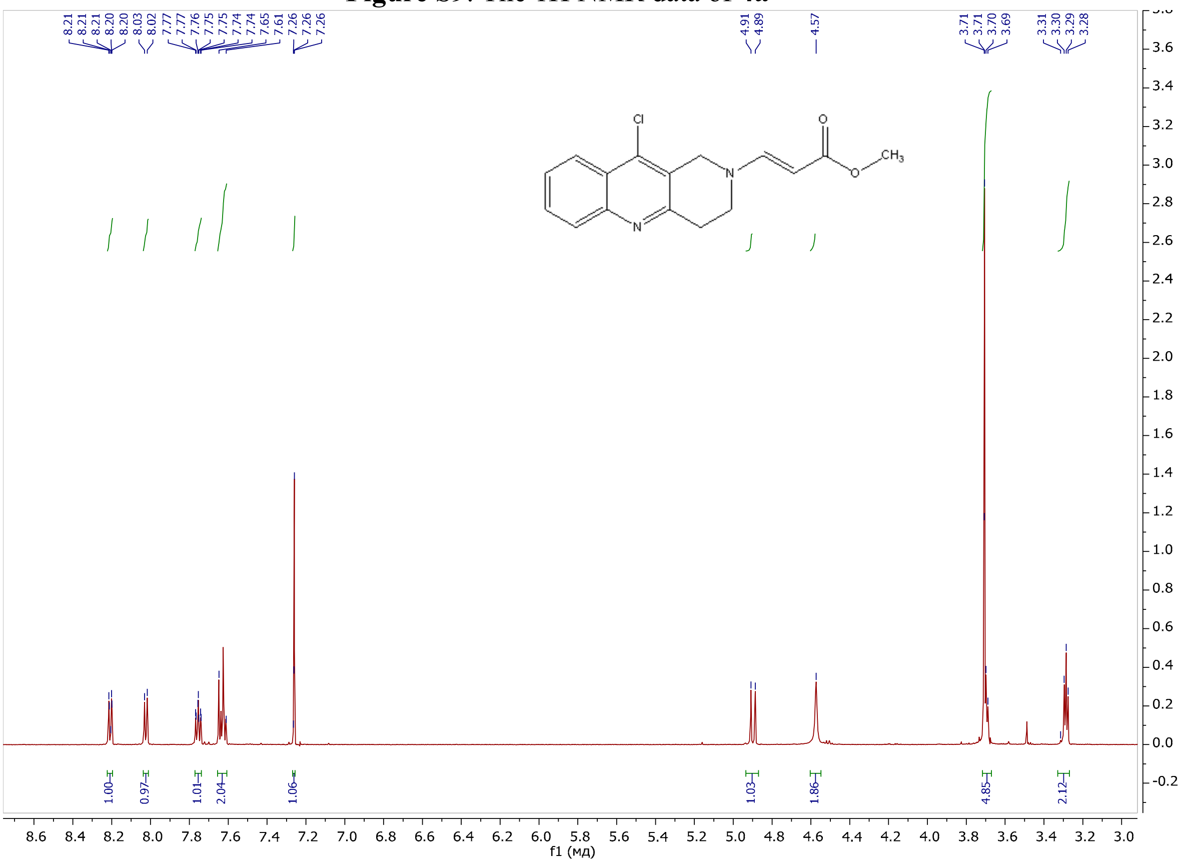

Figure S10. The 13C NMR data of **4a**

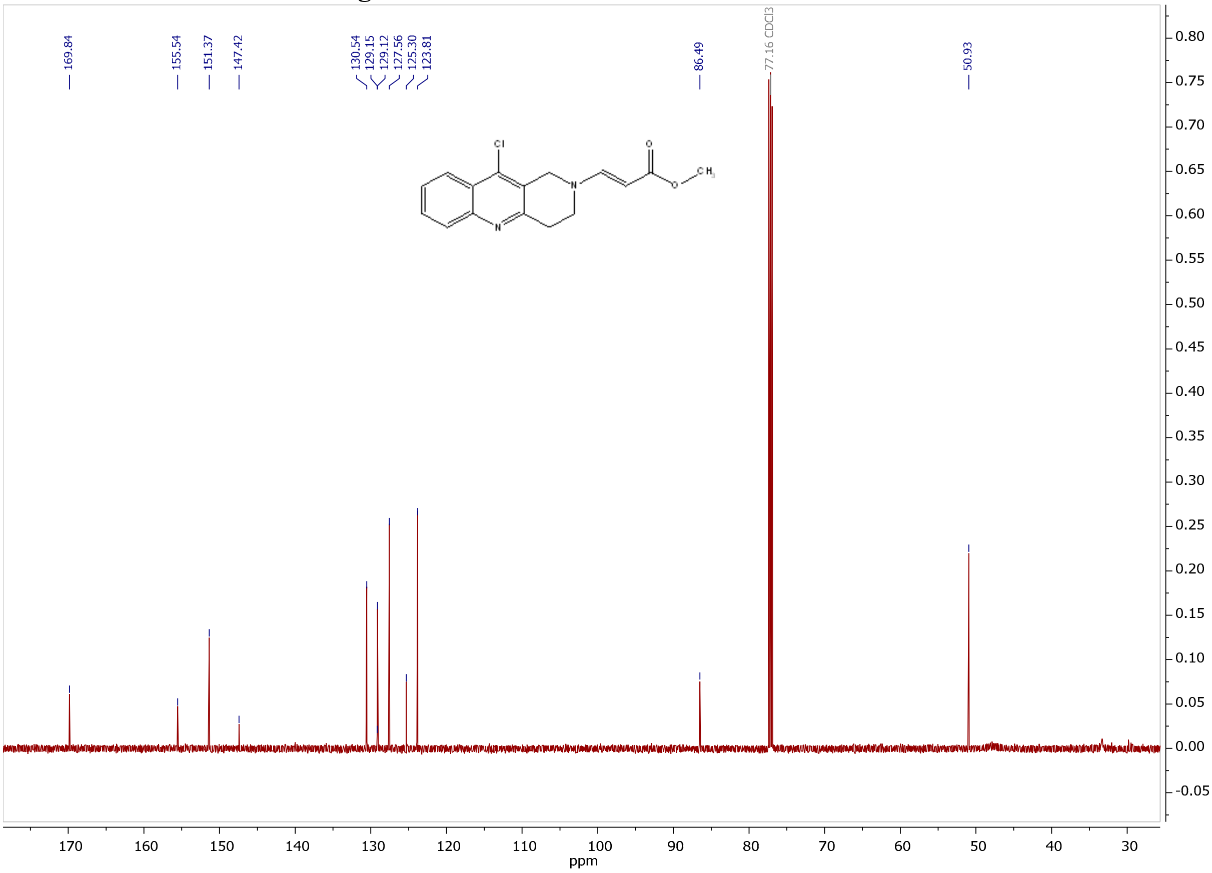

Figure S11. The 1H NMR data of **4b**

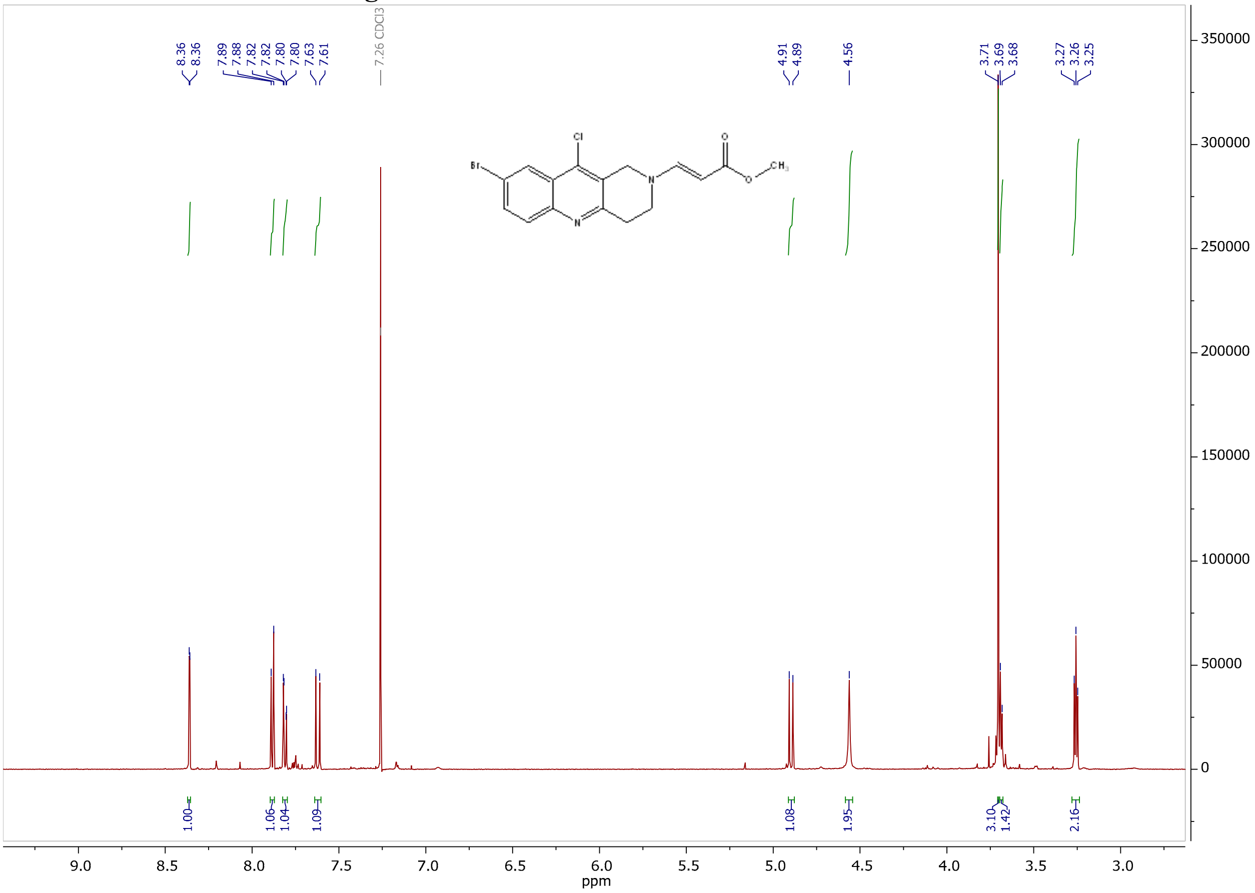

Figure S12. The 13C NMR data of **4b**

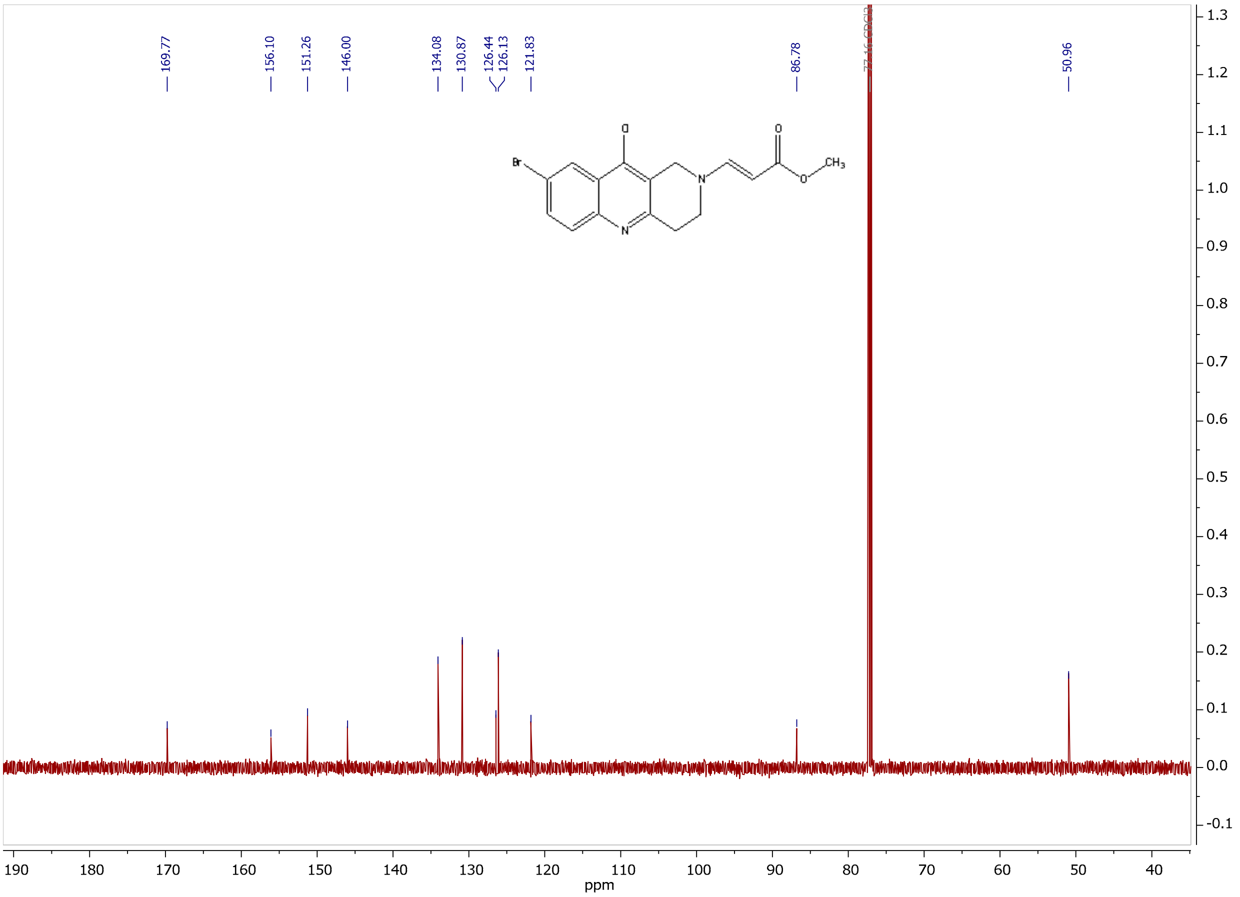

Figure S13. The 1H NMR data of **4c**

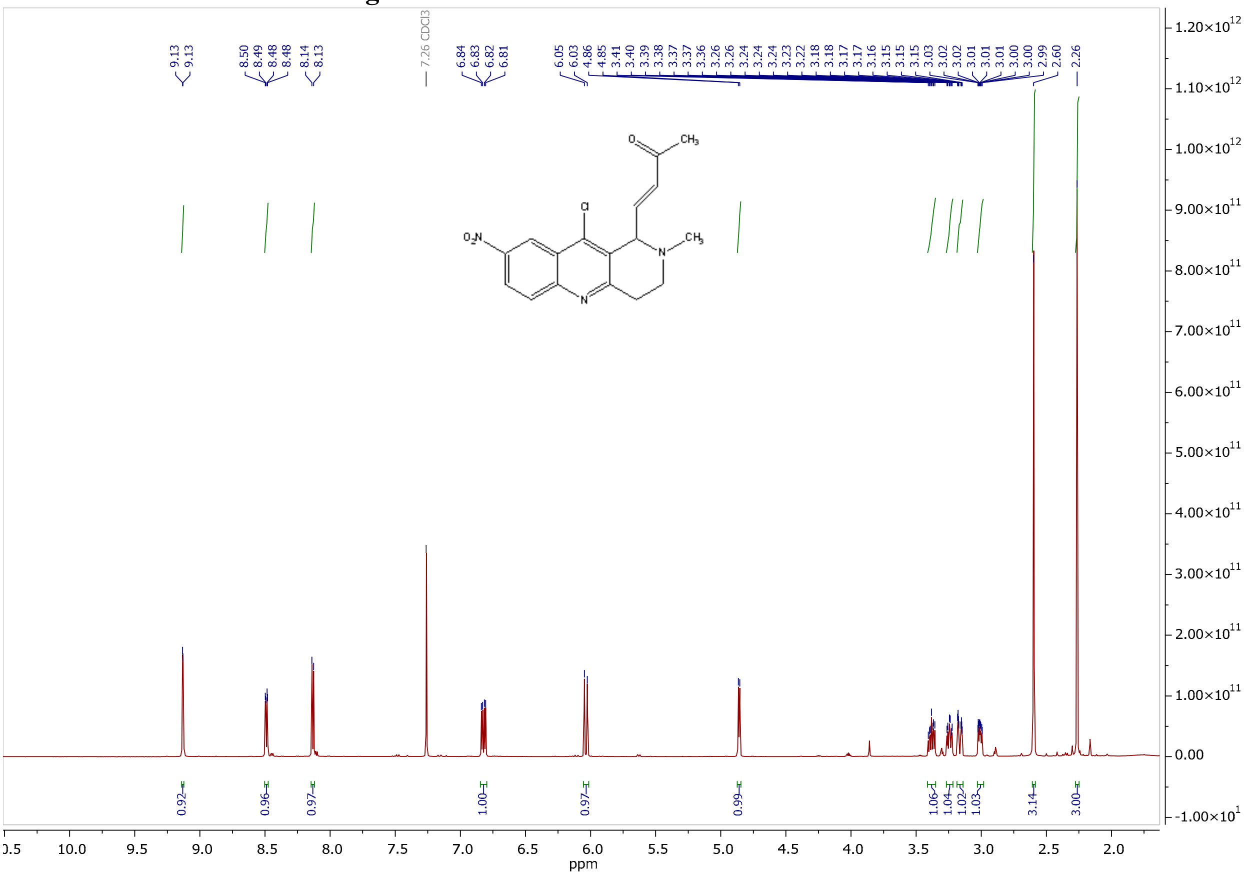

Figure S14. The 13C NMR data of **4c**

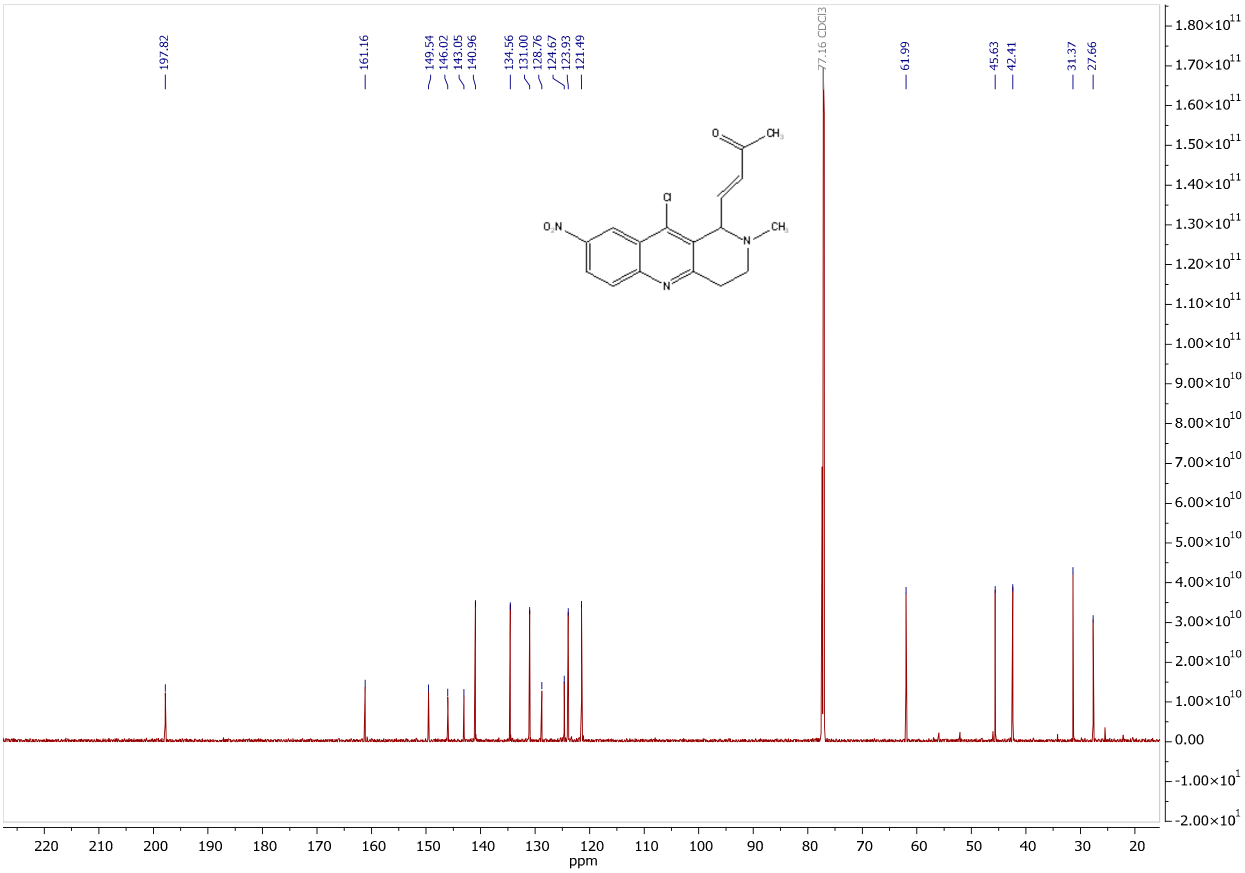

Figure S15. The 1H NMR data of **5a**

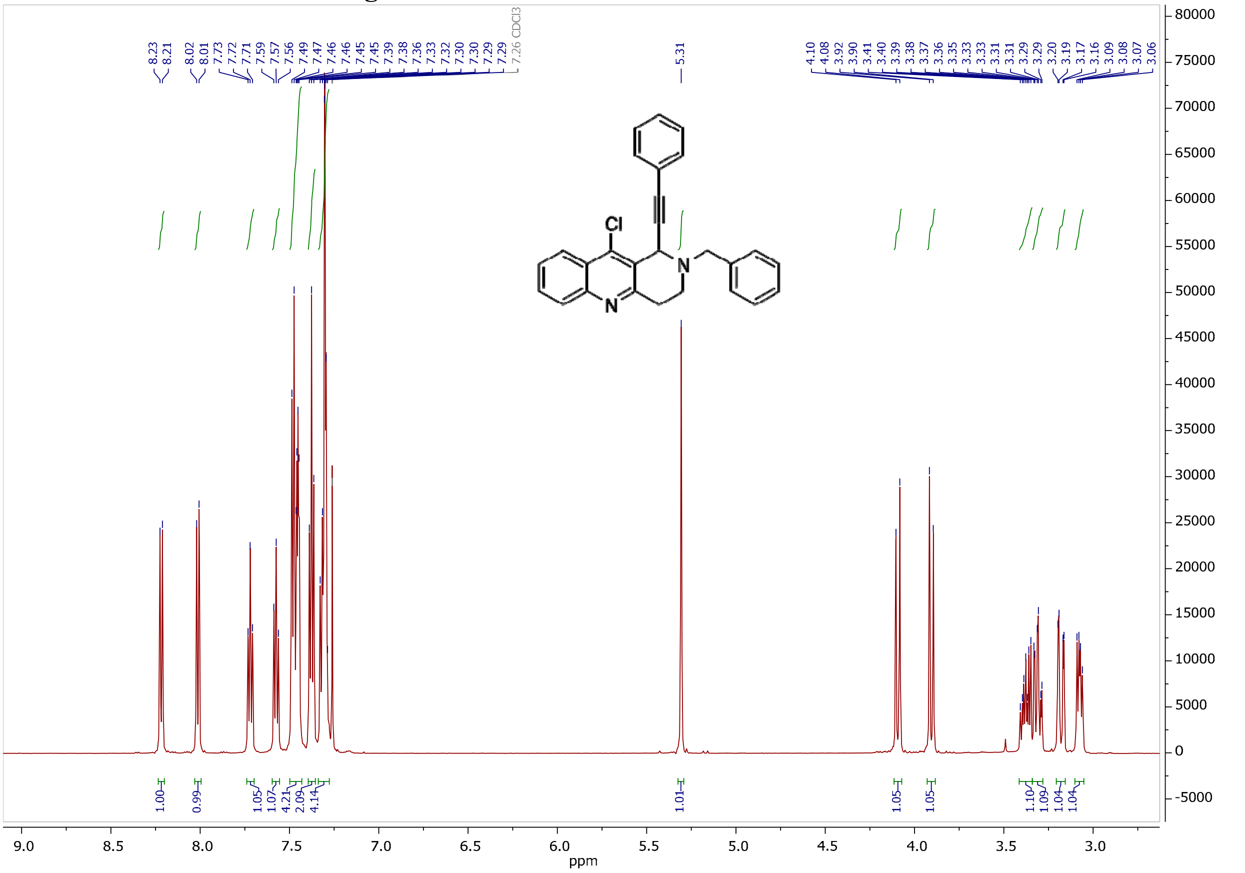

Figure S16. The 13C NMR data of **5a**

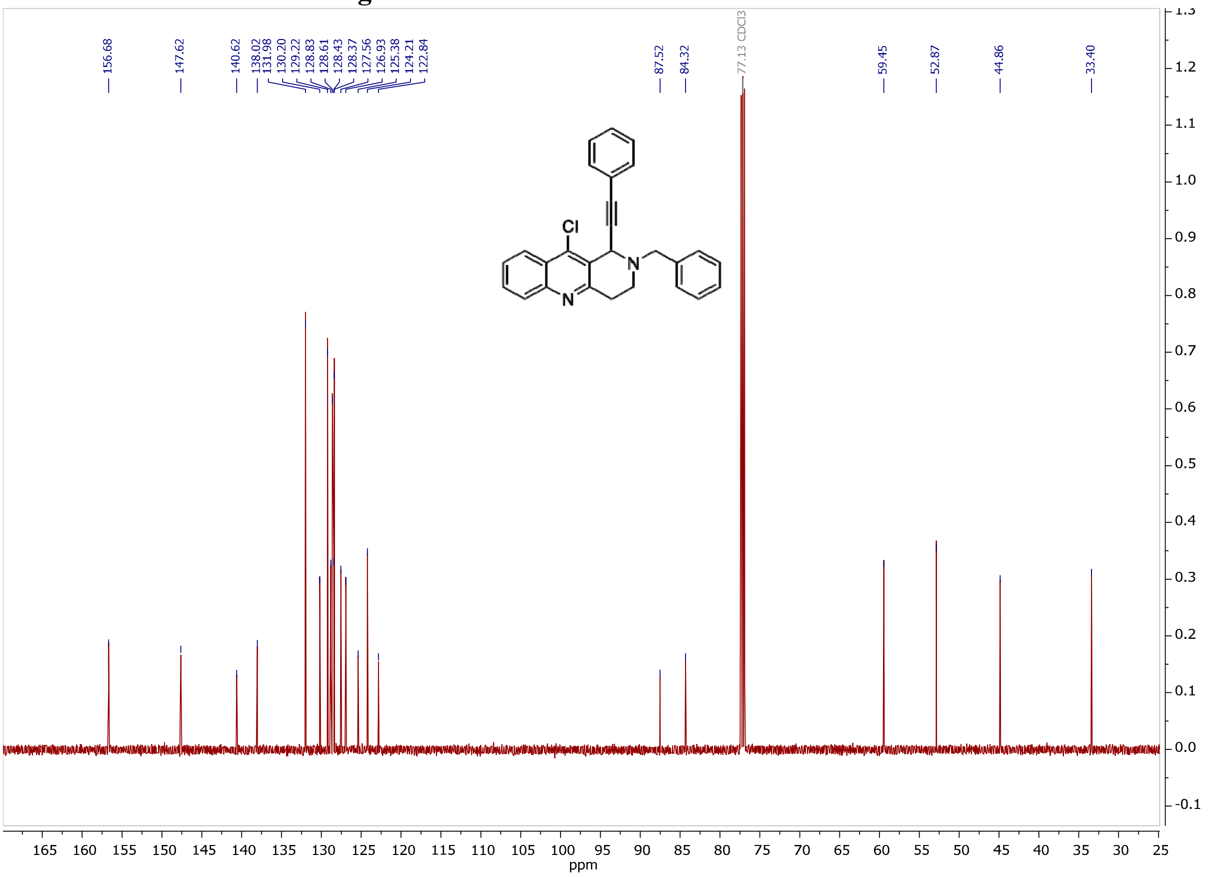

Figure S17. The 1H NMR data of **5b**

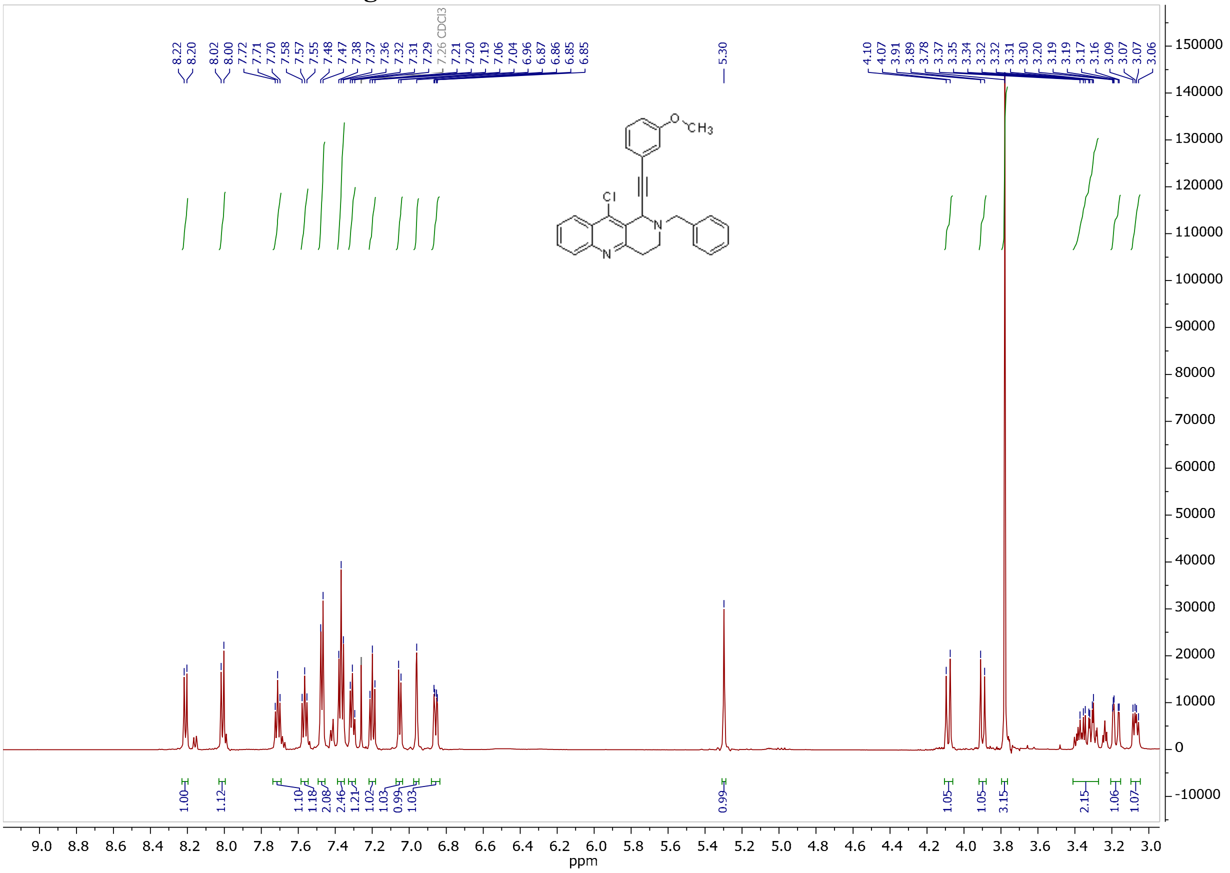

Figure S18. The 13C NMR data of **5b**

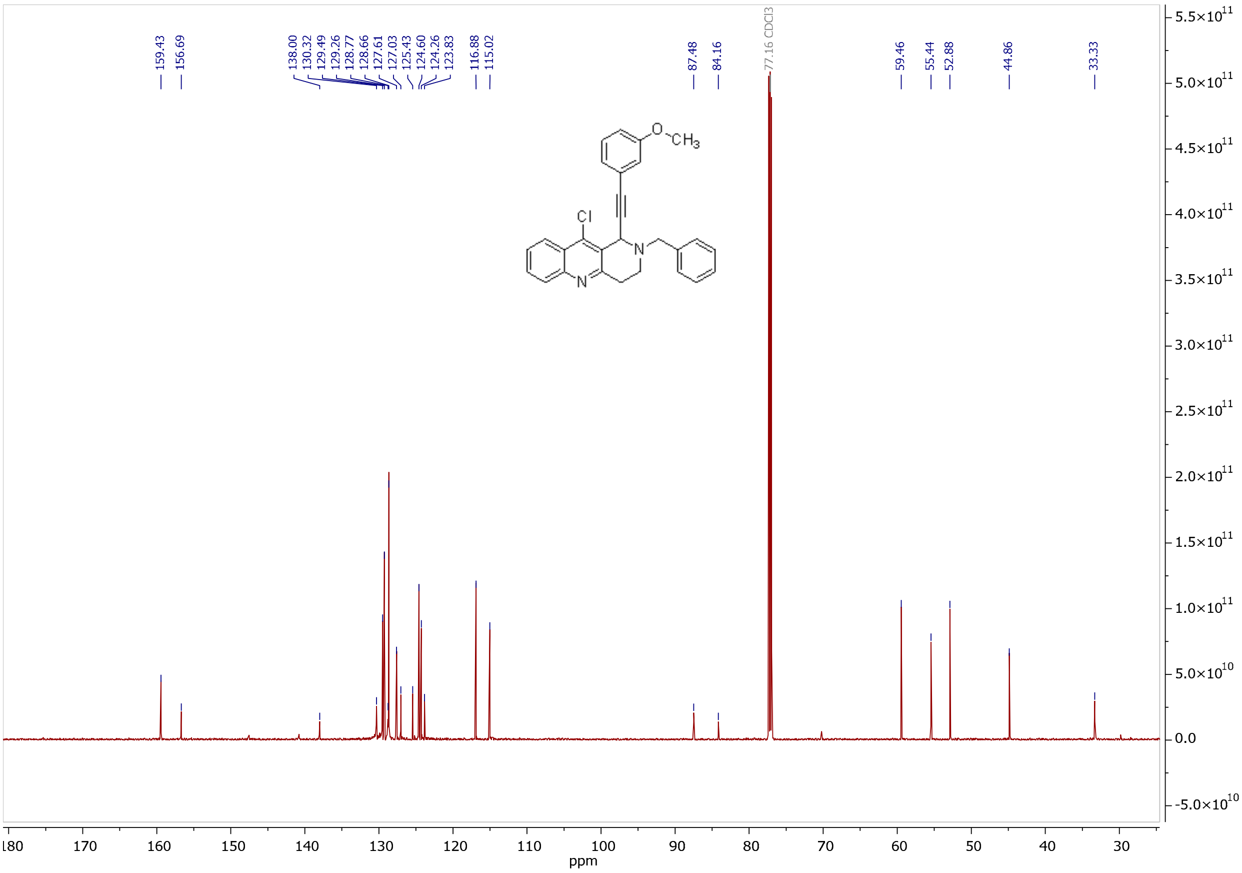

Figure S19. The 1H NMR data of **5c**

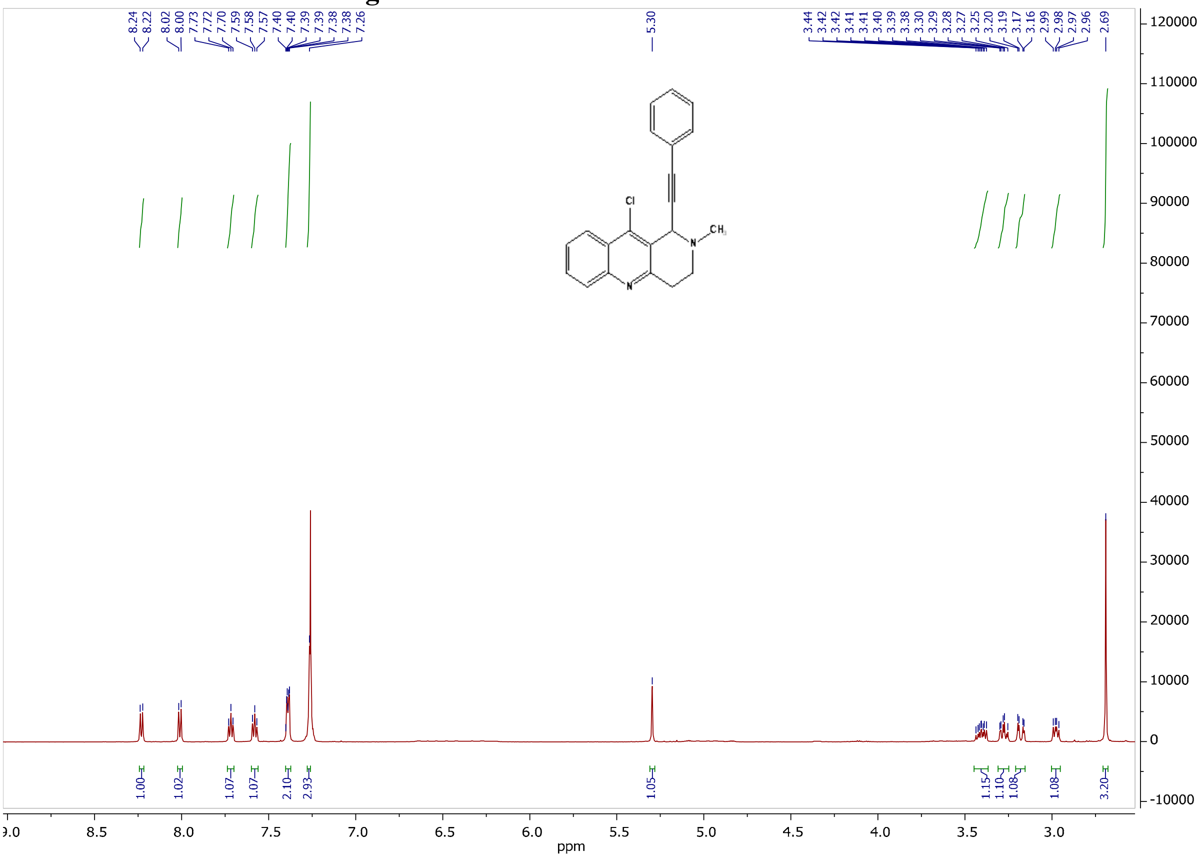

Figure S20. The 1H NMR data of **5d**

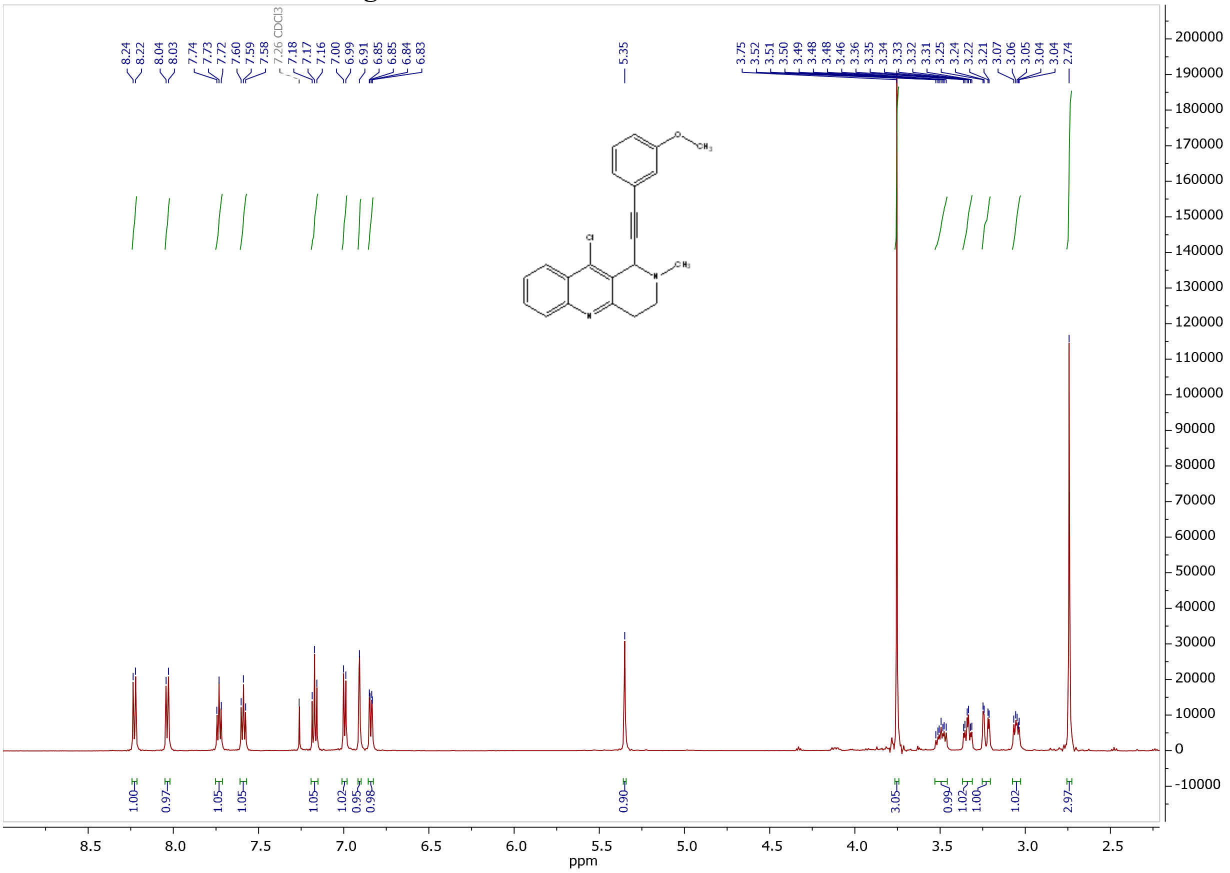

Figure S21. The 13C NMR data of **5d**

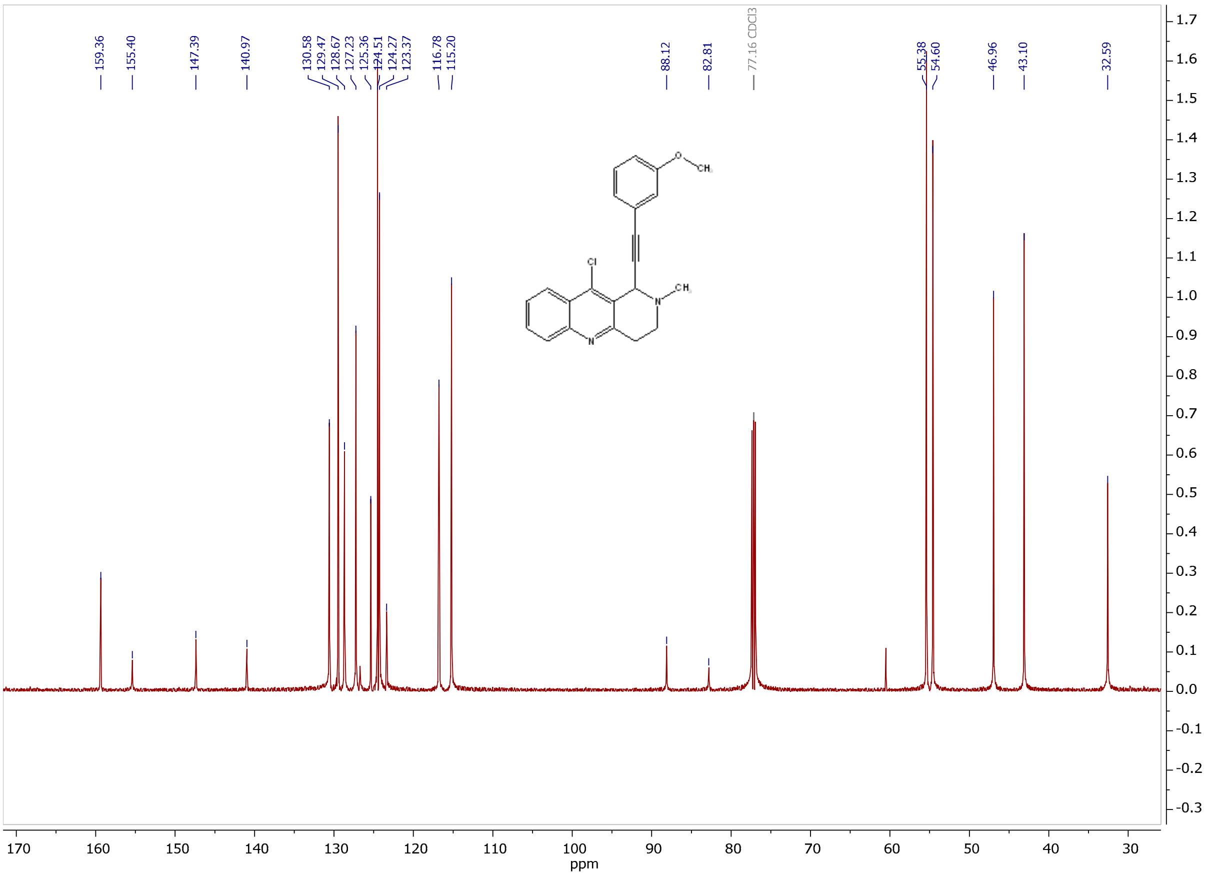

Figure S22. The 1H NMR data of **5e**

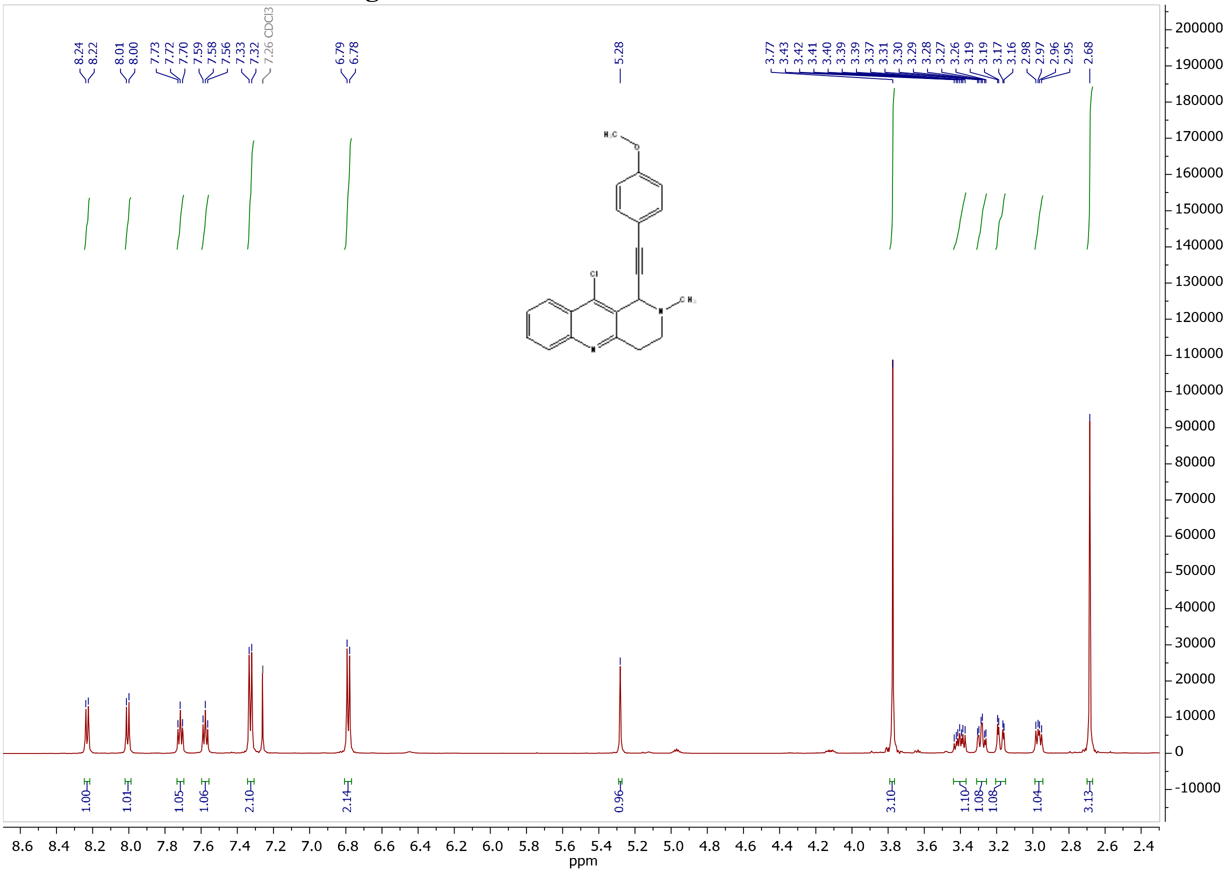

Figure S23. The 13C NMR data of **5e**

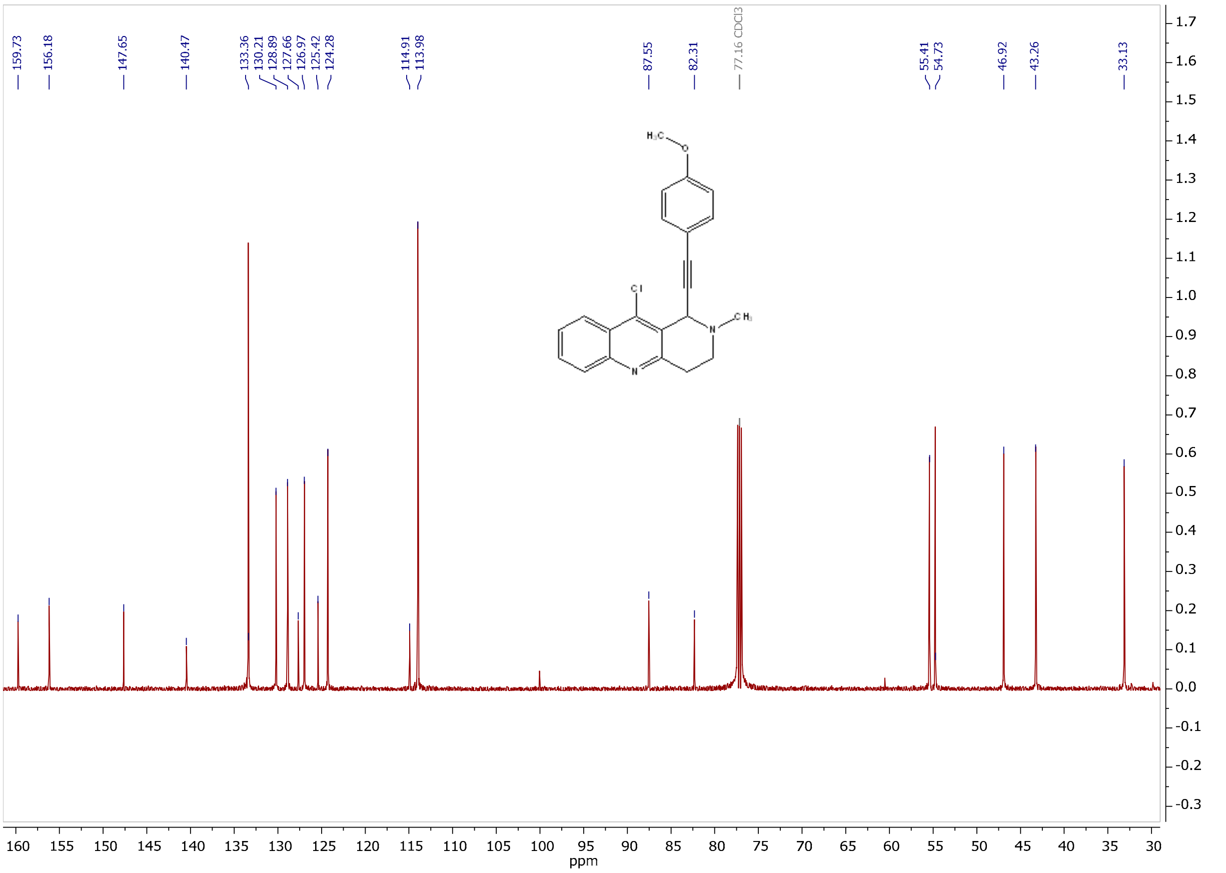

Figure S24. The 1H NMR data of **5f**

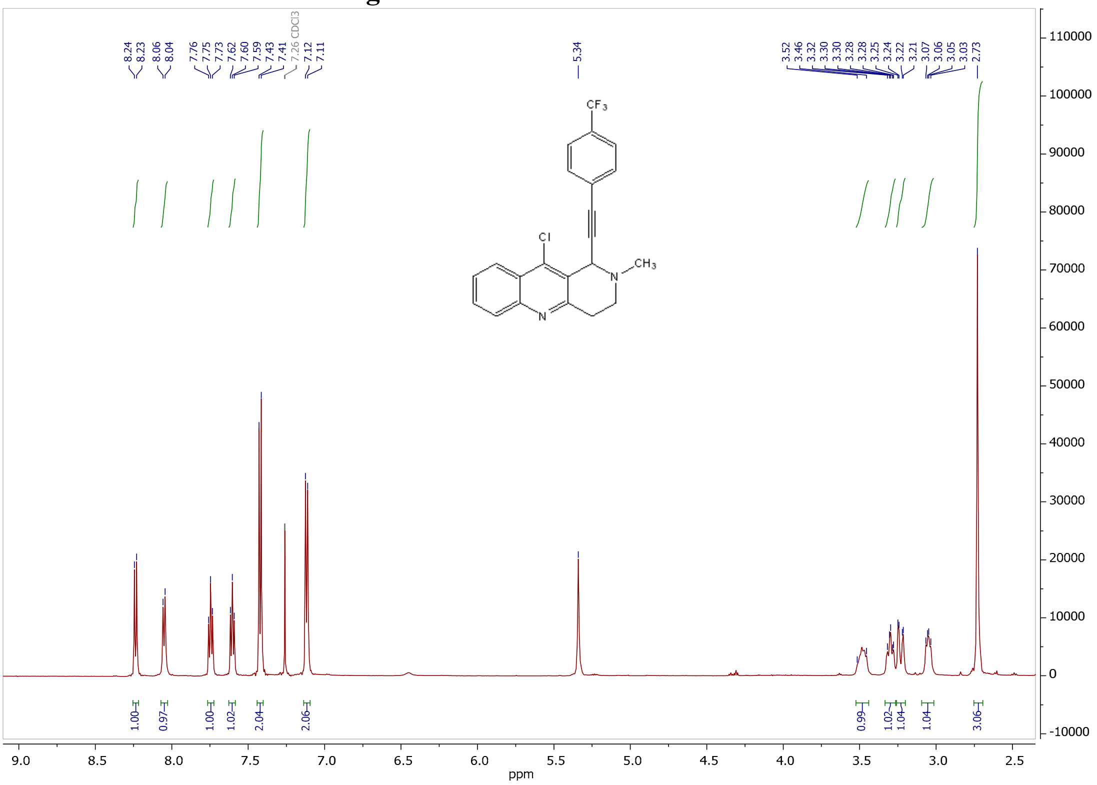

Figure S25. The 13C NMR data of **5f**

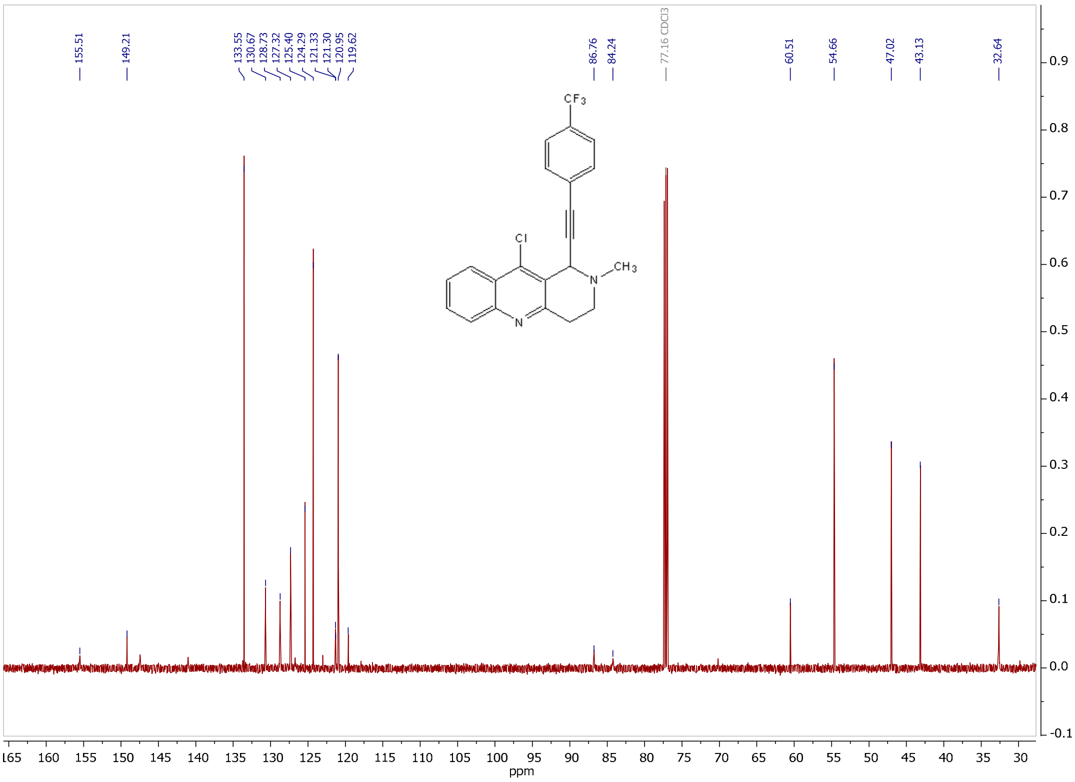

Figure S26. The 1H NMR data of **5g**

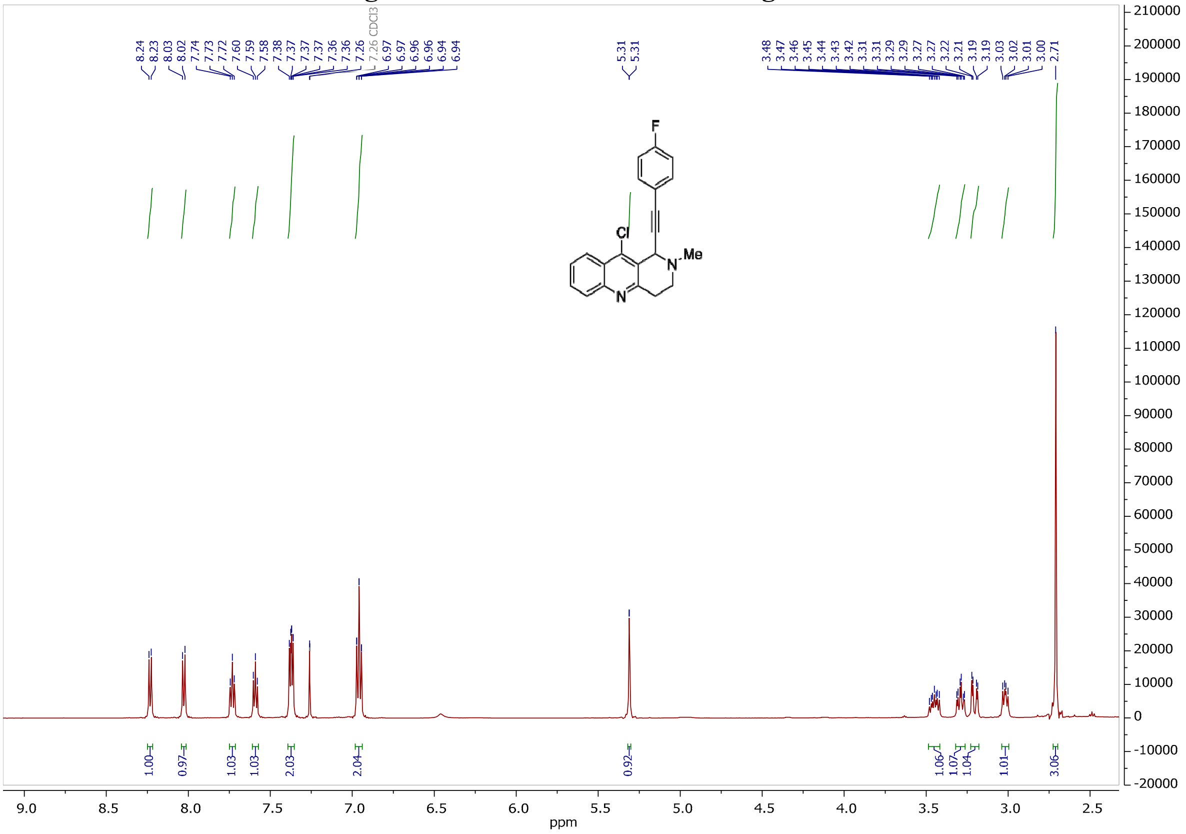

Figure S27. The 13C NMR data of **5g**

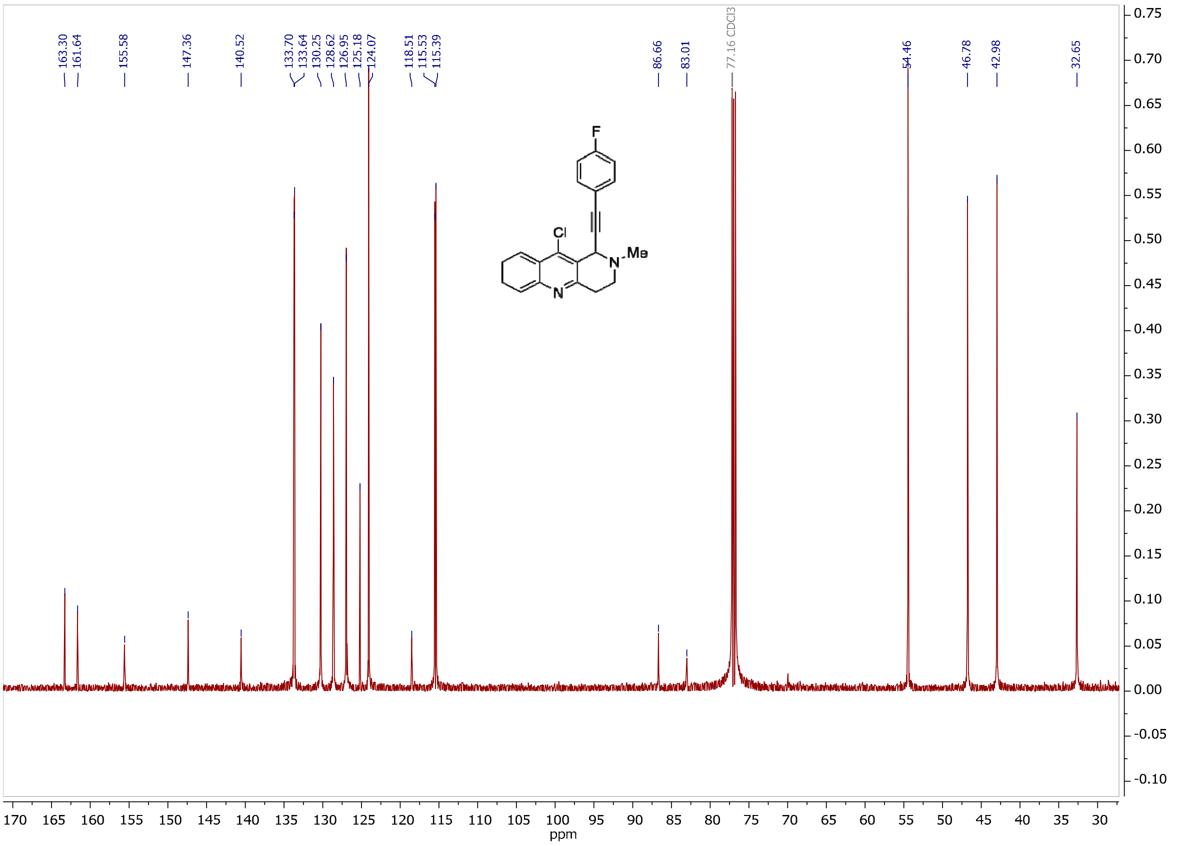

Figure S28. The 1H NMR data of **5h**

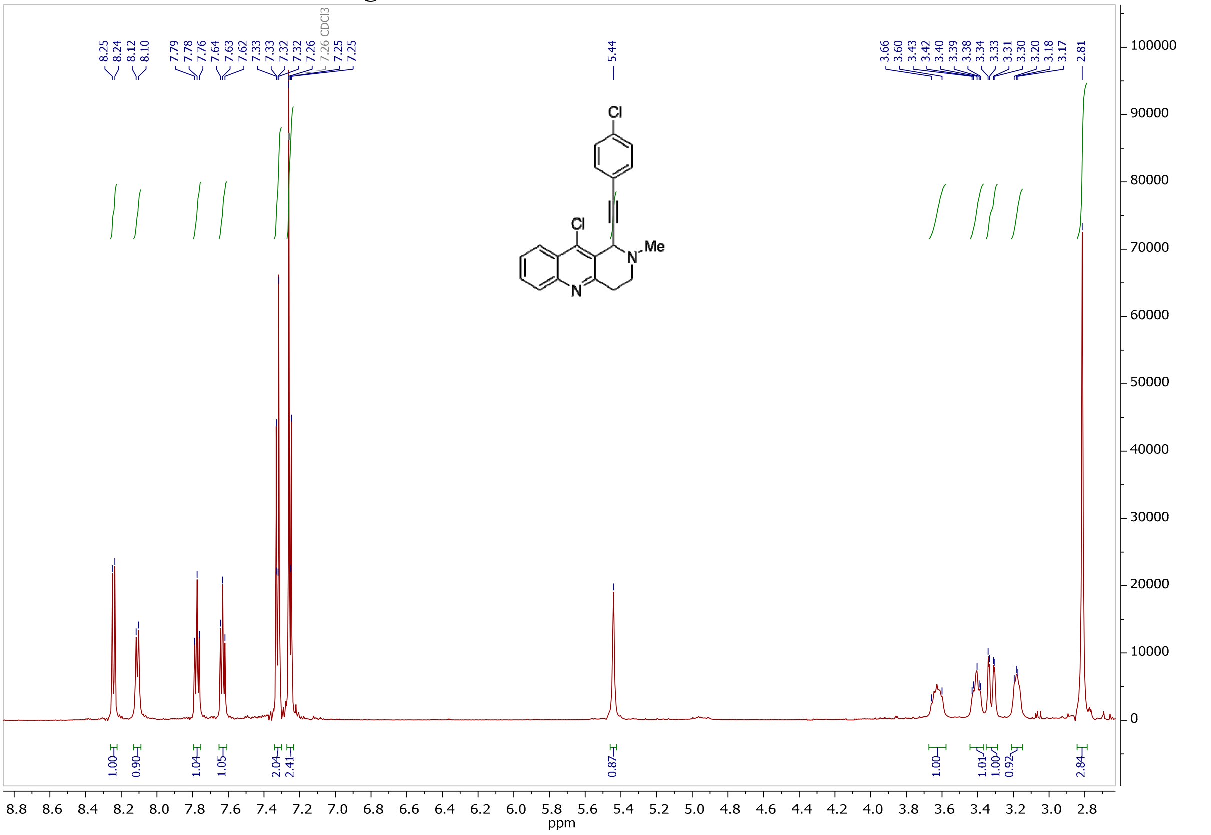

Figure S29. The 1H NMR data of **6a**

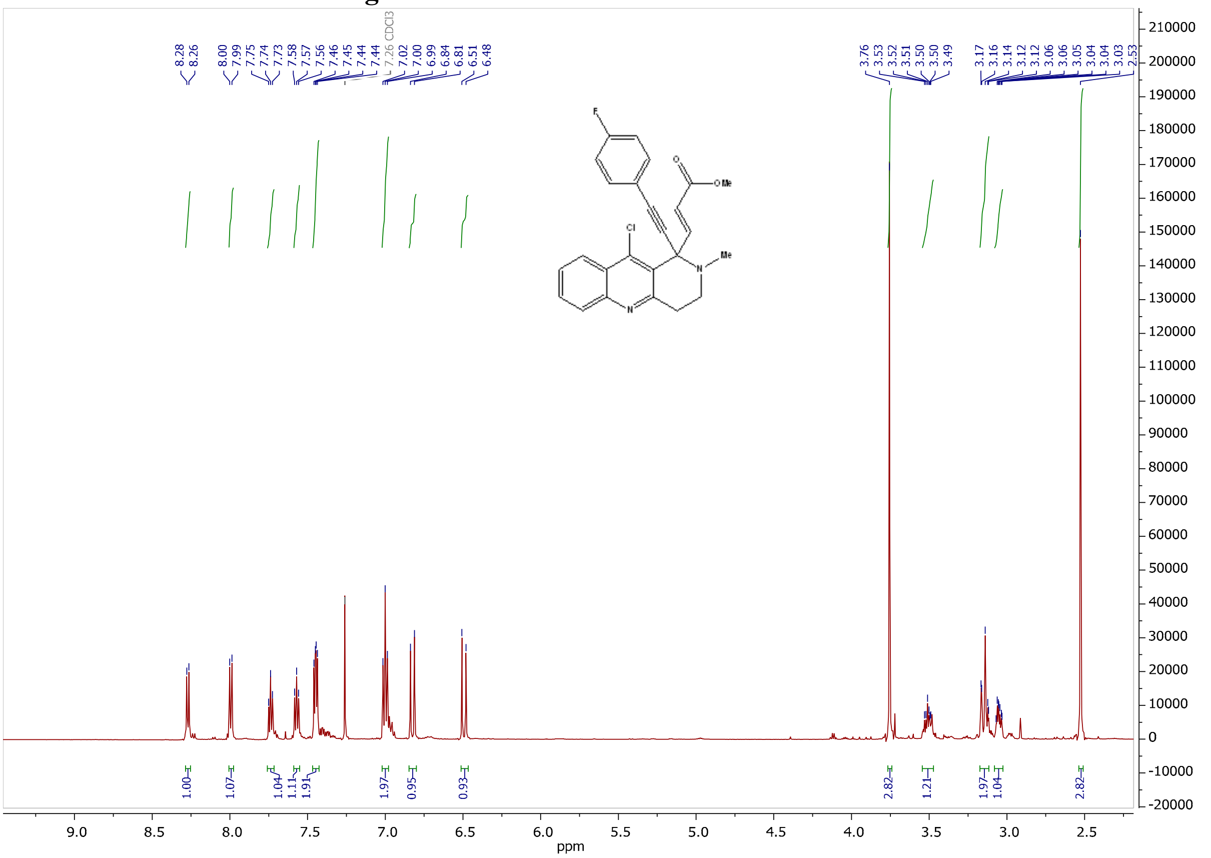

Figure S30. The 13C NMR data of **6a**

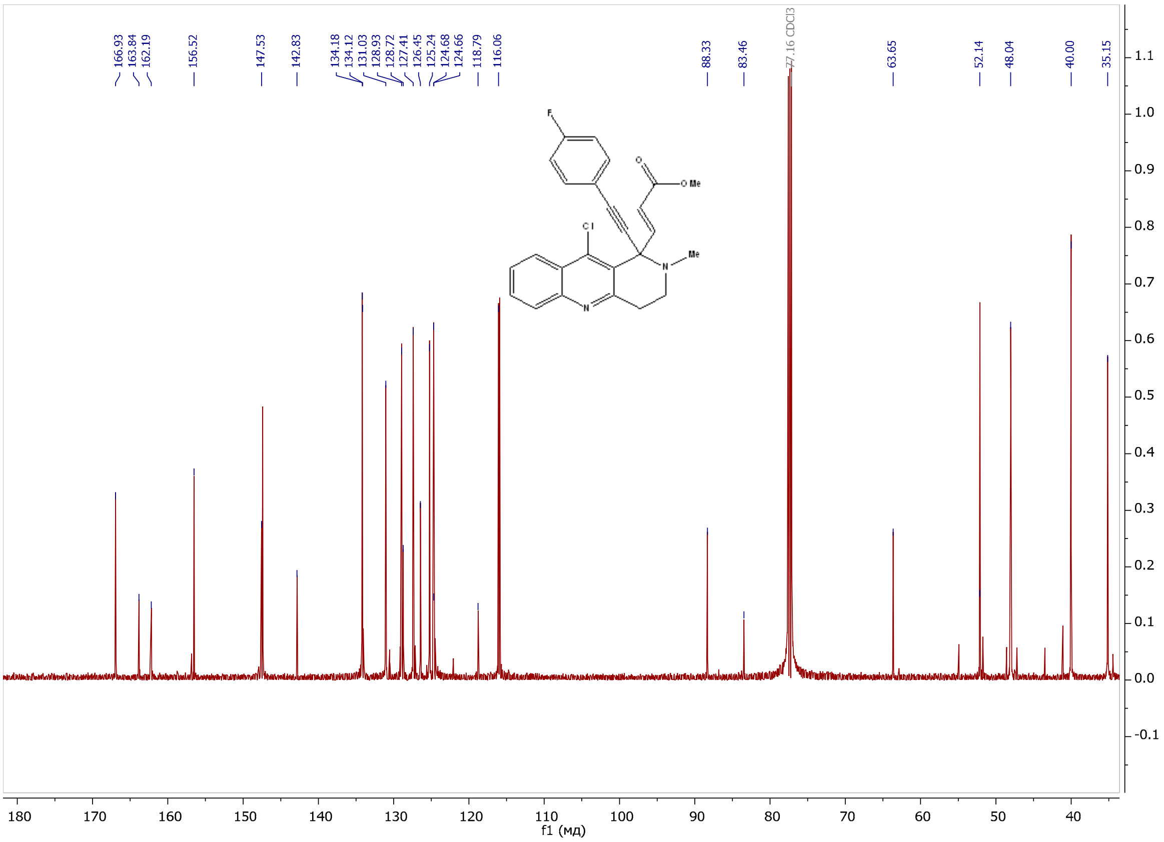

Figure S31. The 1H NMR data of **6b**

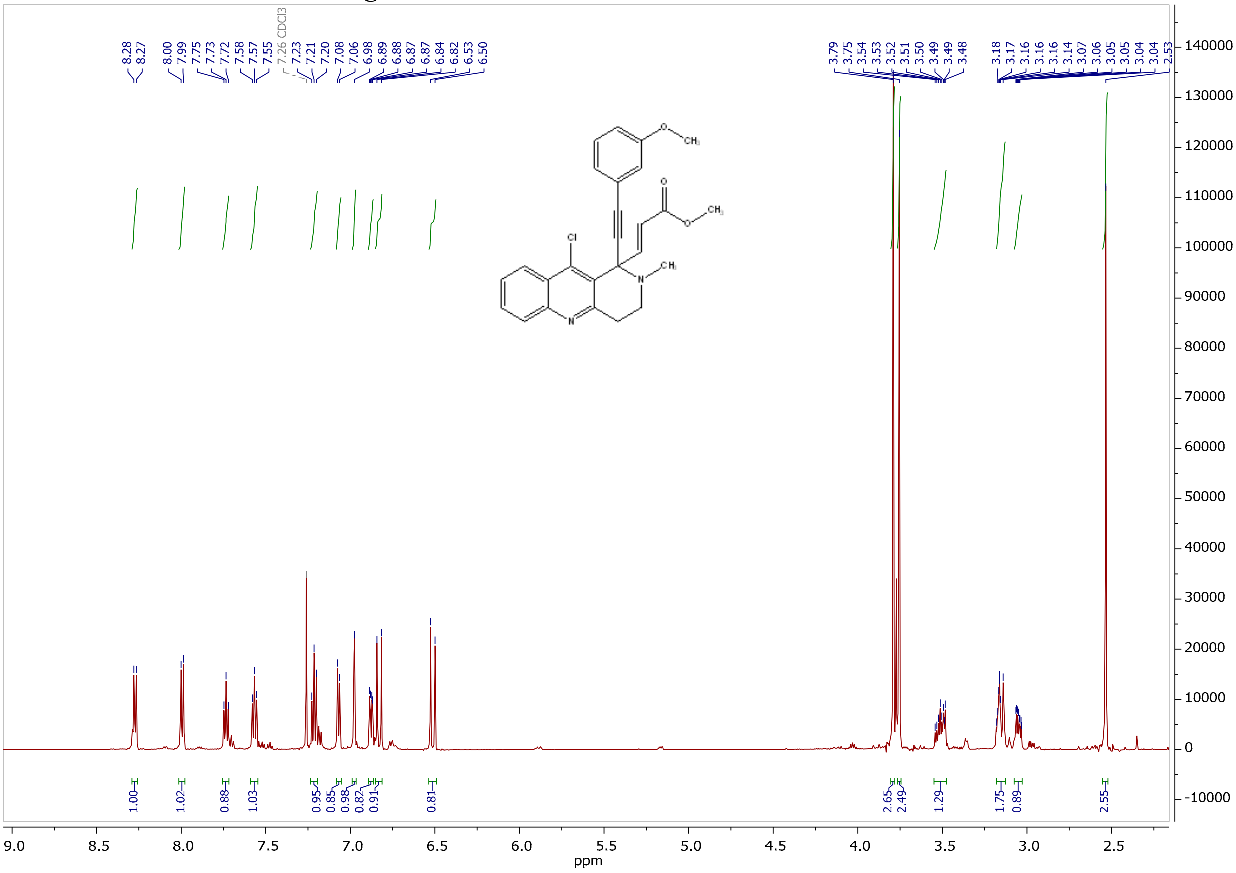

Figure S32. The 13C NMR data of **6b**

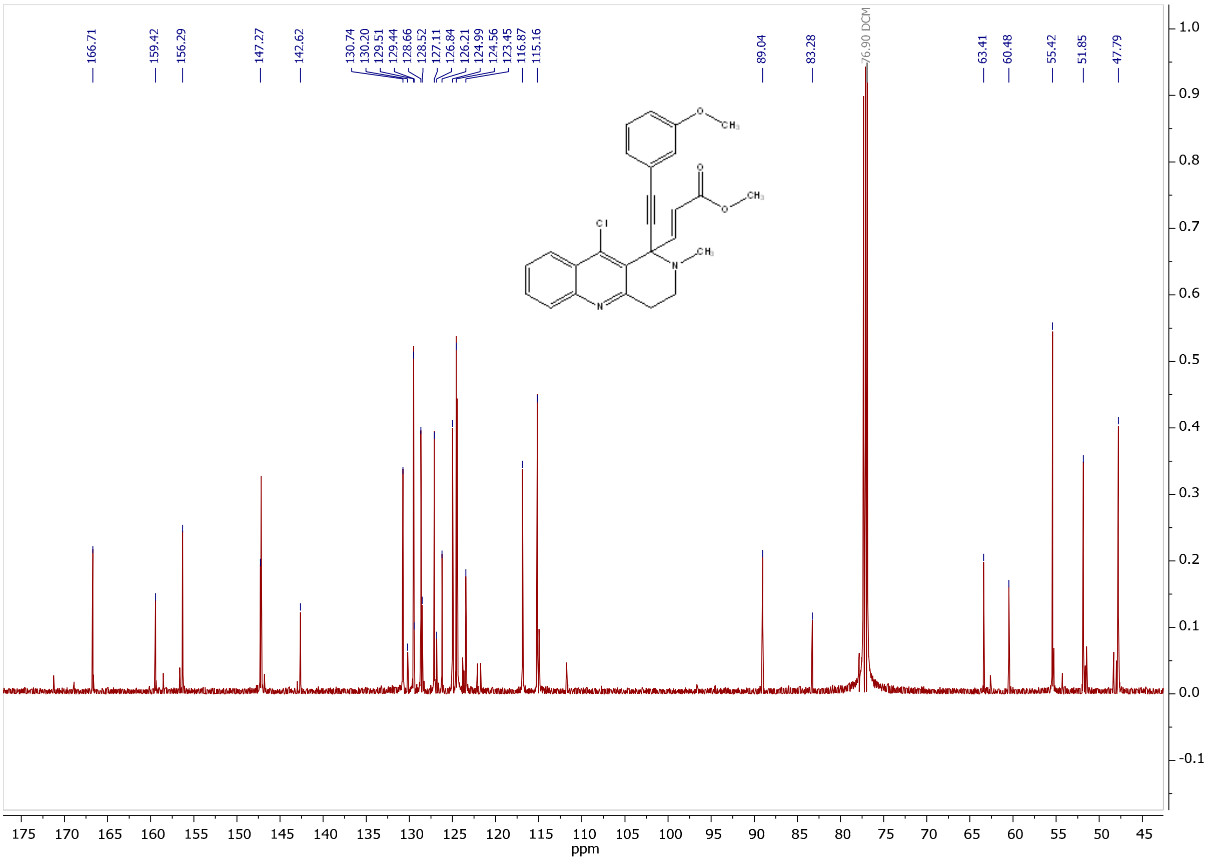

Figure S33. The 1H NMR data of **6c**

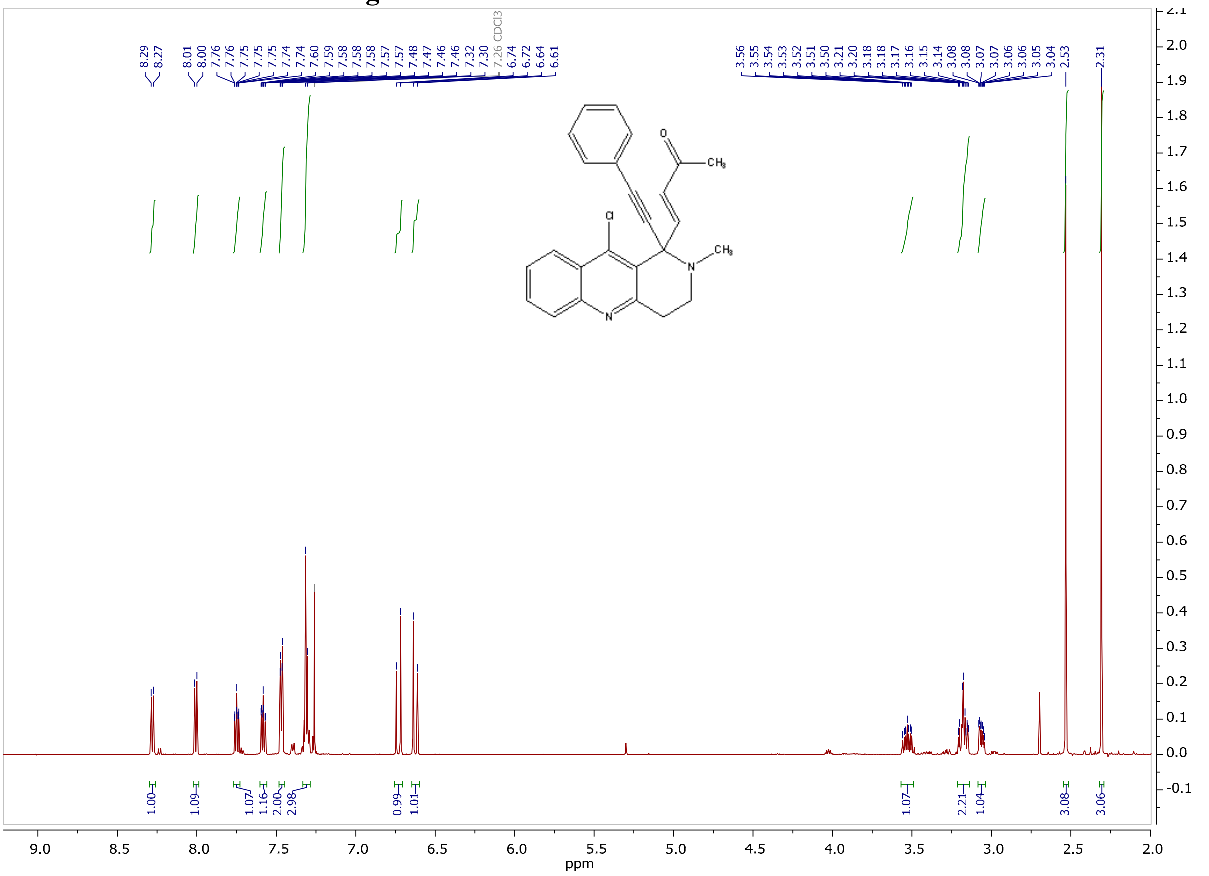

Figure S34. The 13C NMR data of **6c**

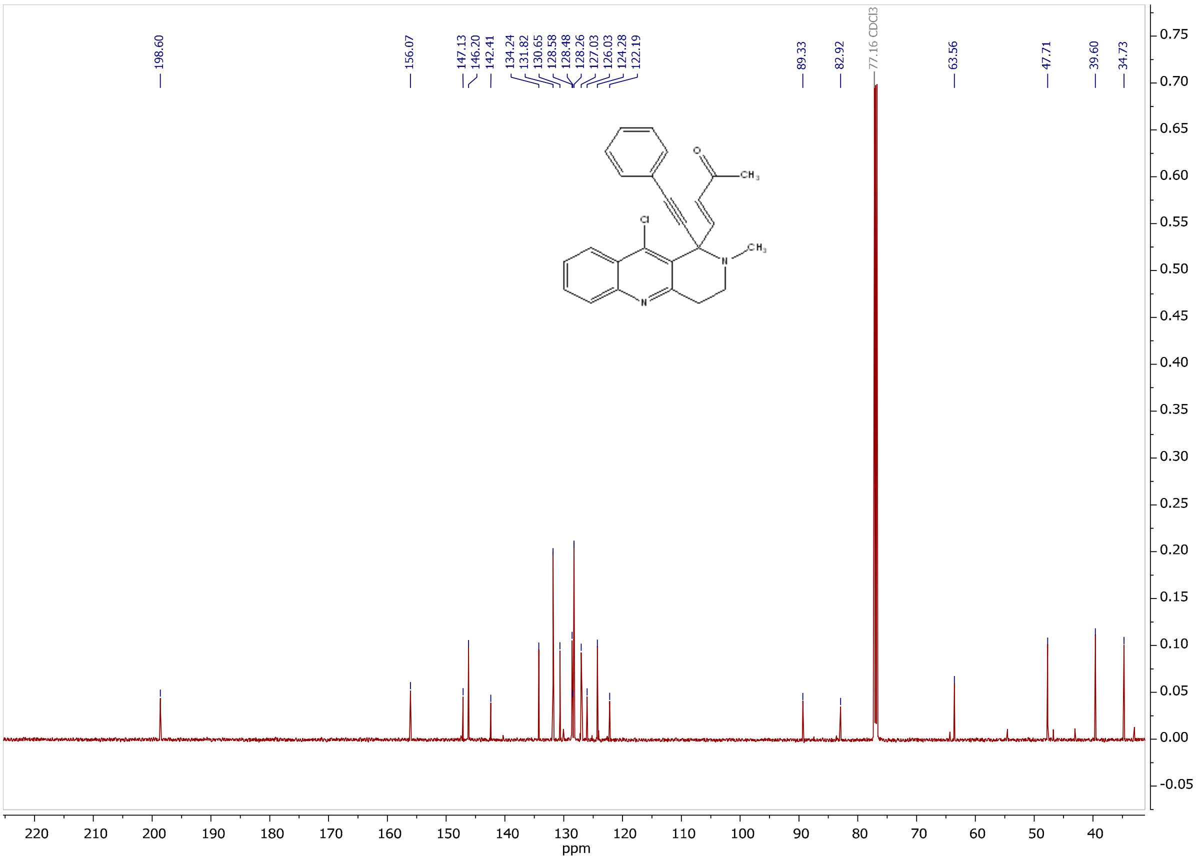

Figure S35. The 1H NMR data of **6d**

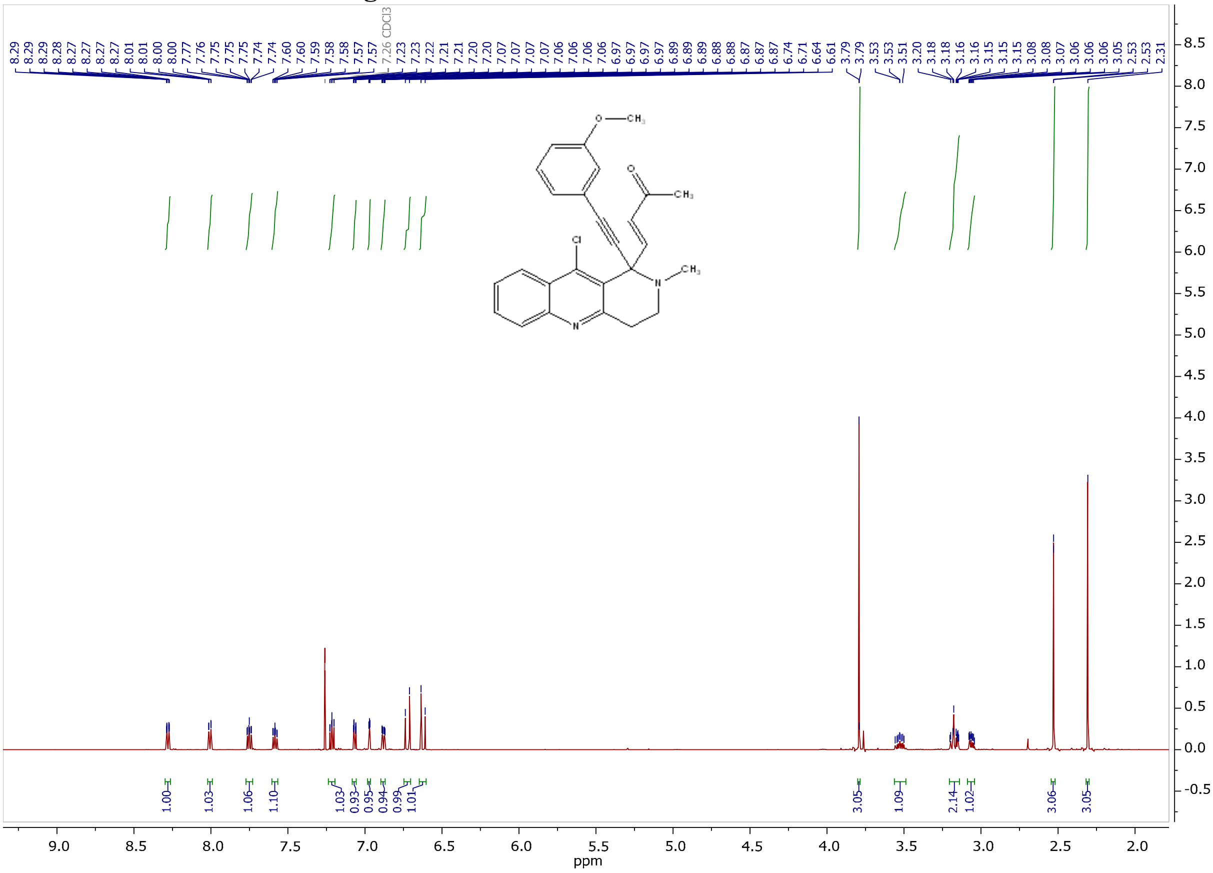

Figure S36. The 13C NMR data of **6d**

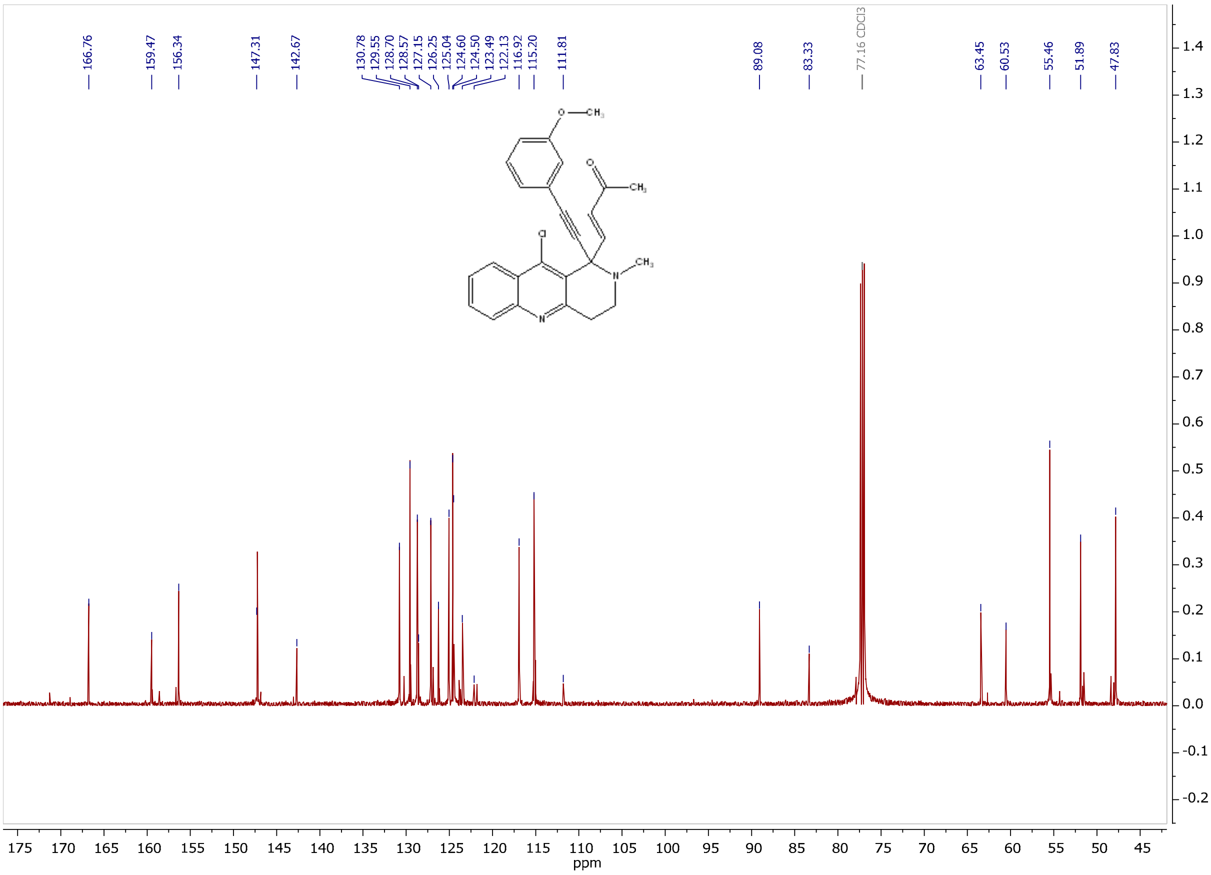

Figure S37. The 1H NMR data of **6e**

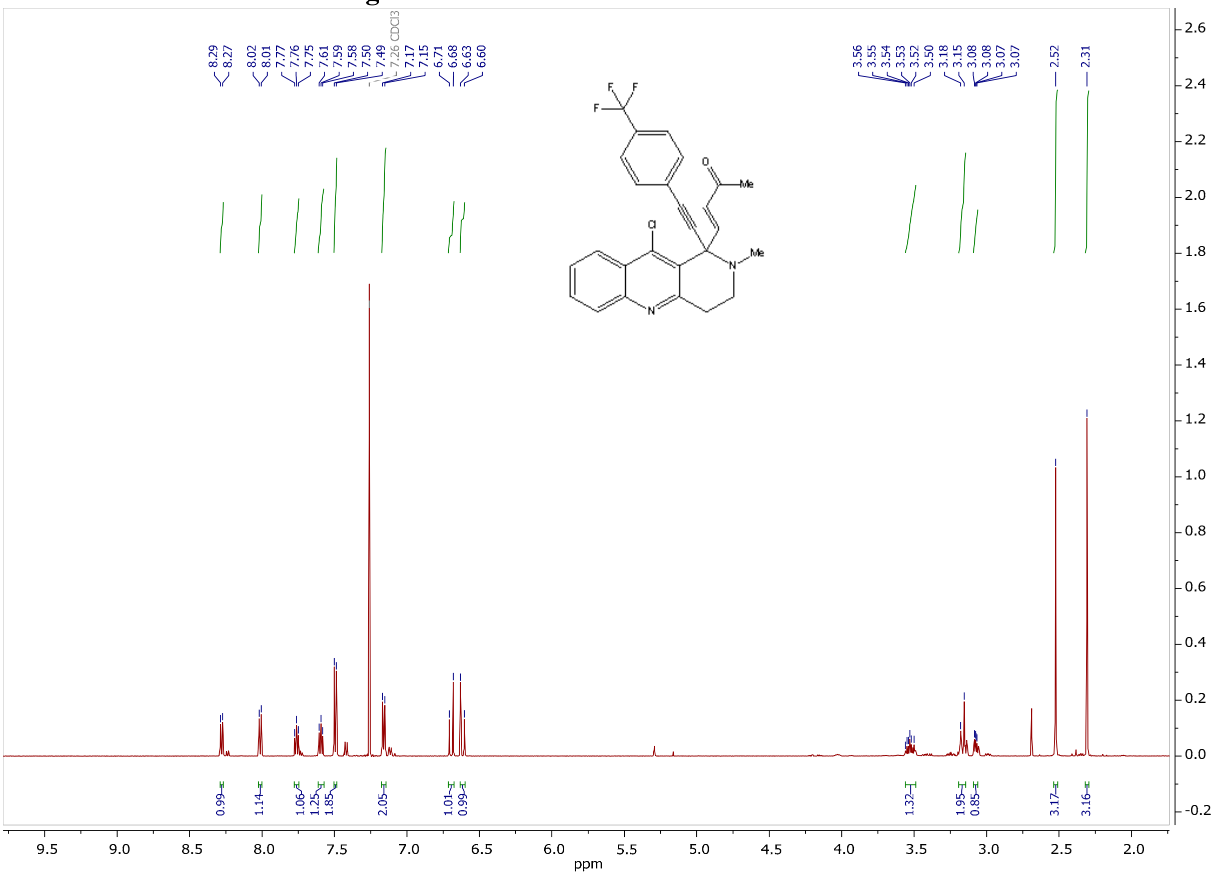

Figure S38. The 1H NMR data of **7**

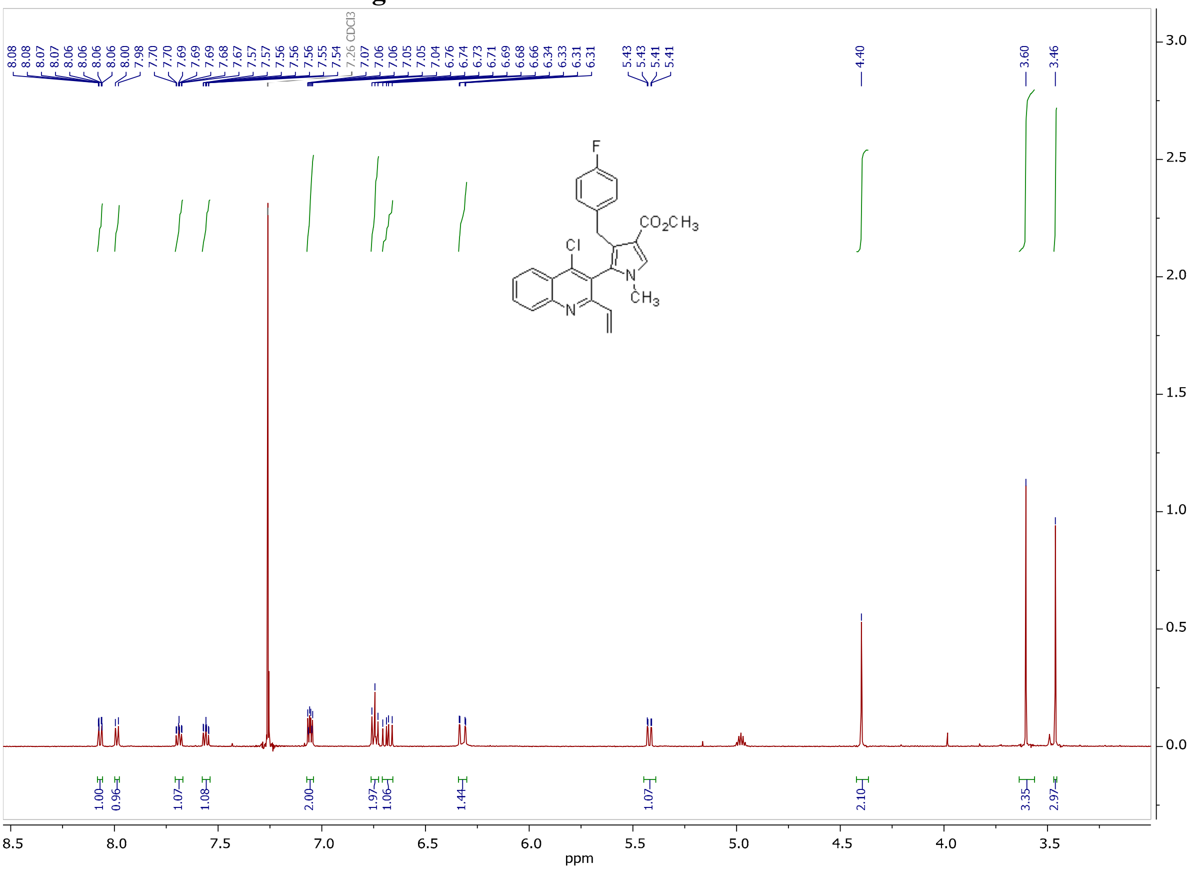

Figure S39. The 13C NMR data of **7**

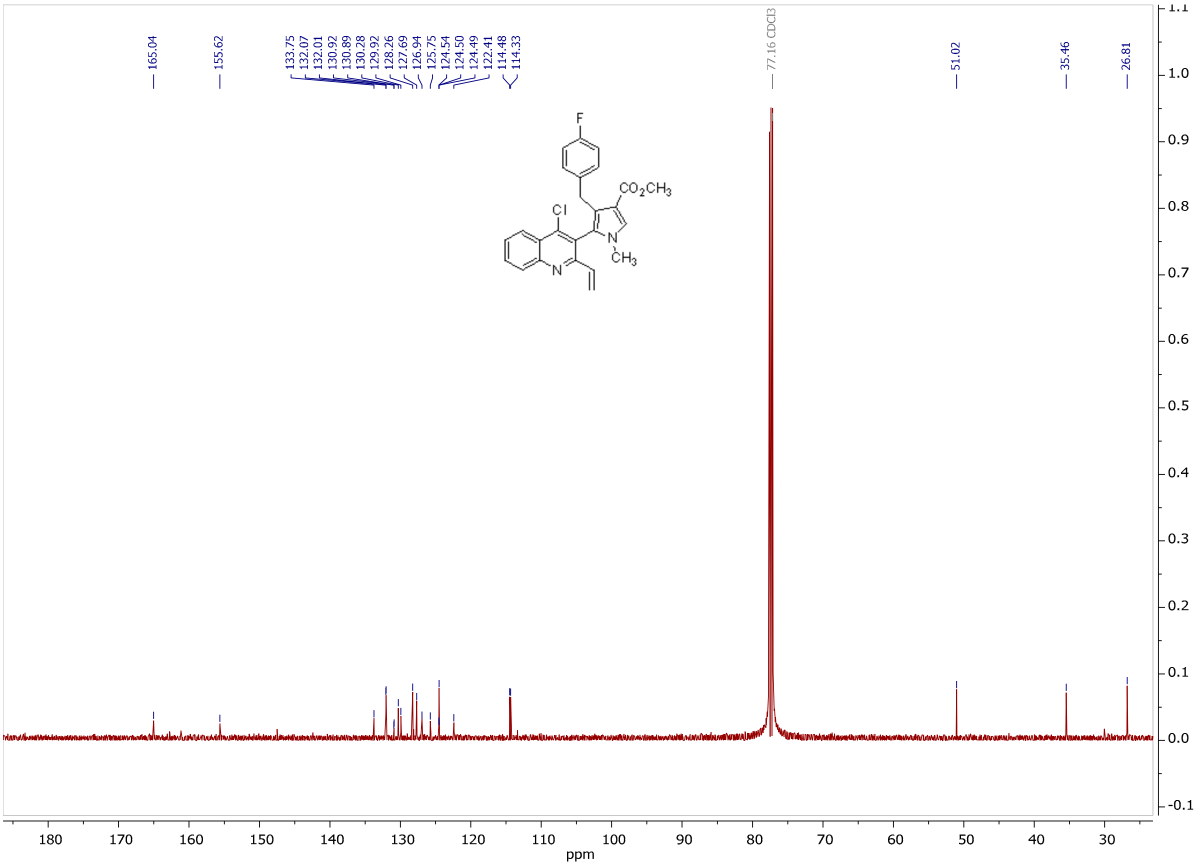

Figure S40. The 1H NMR data of **8a**

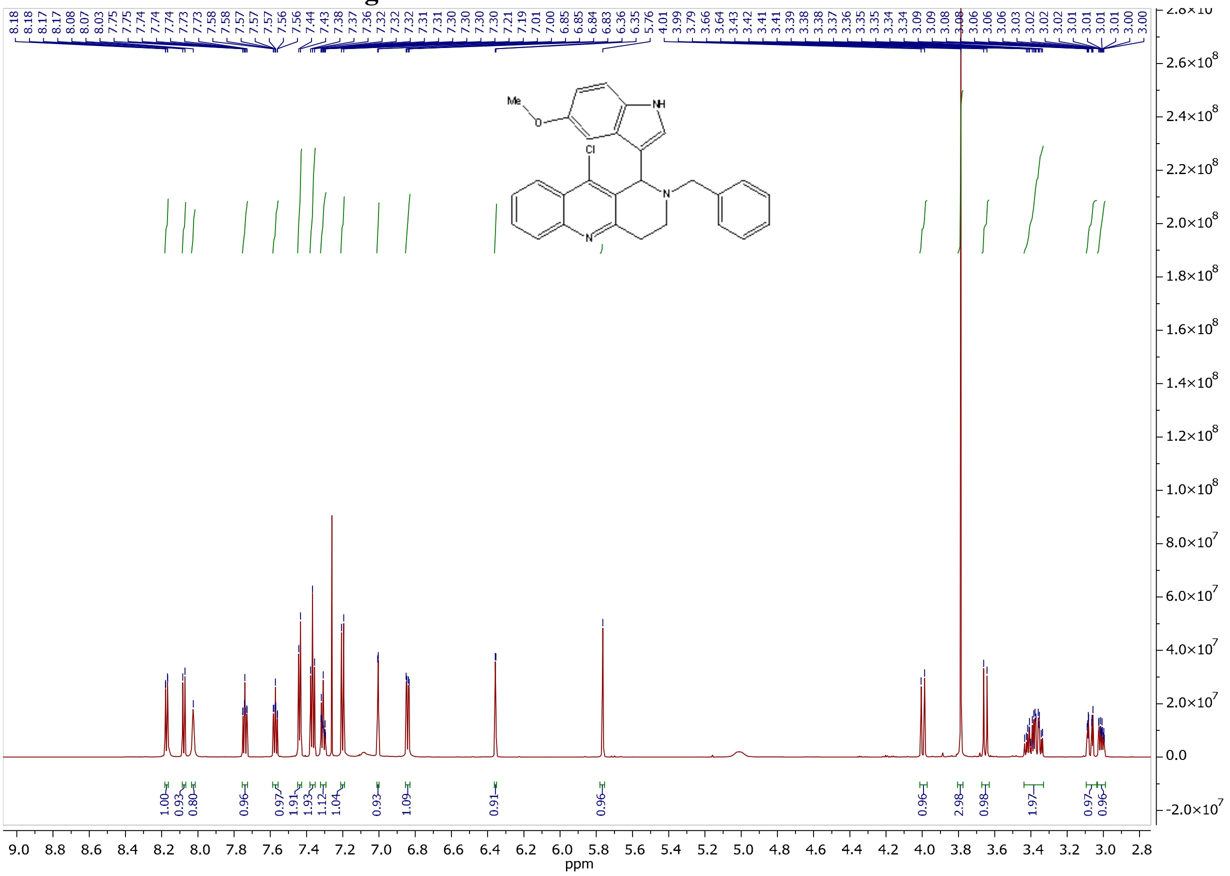

Figure S41. The 13C NMR data of **8a**

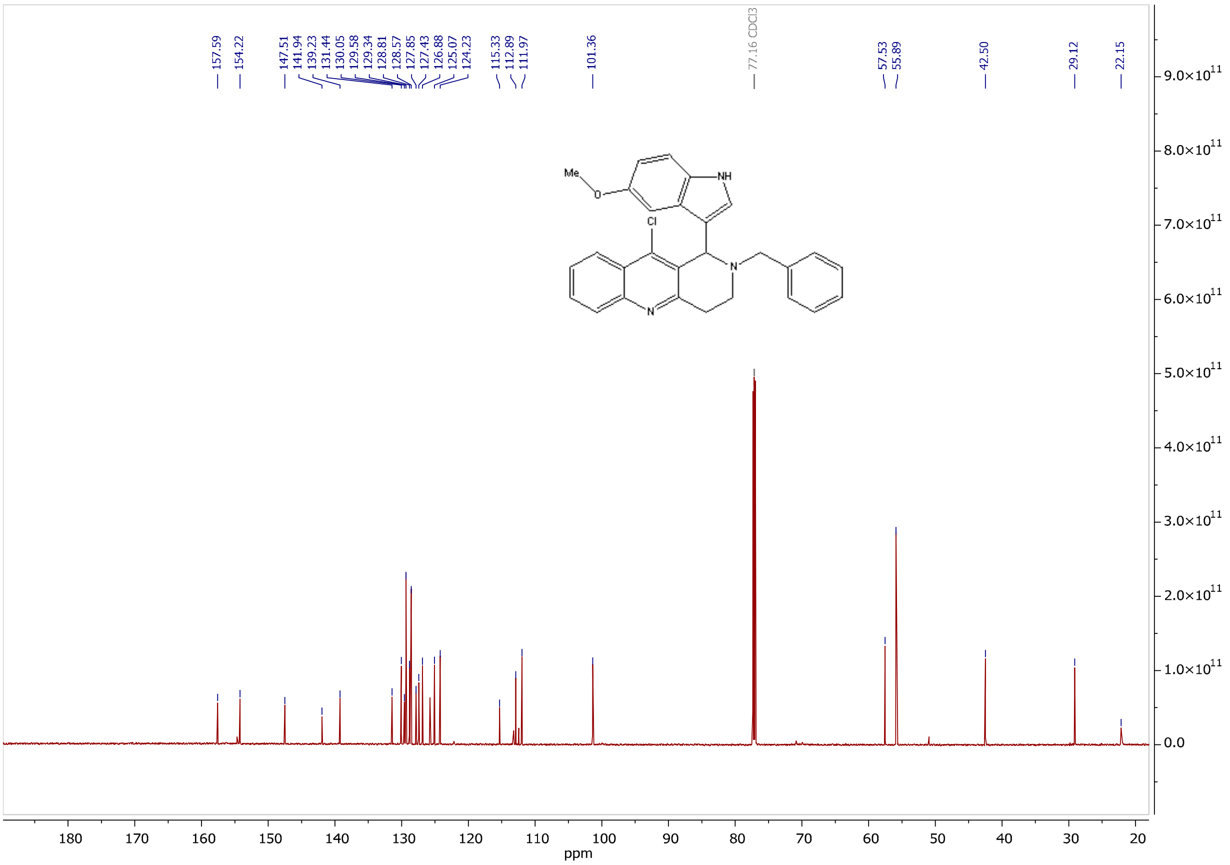

Figure S42. The 1H NMR data of **8b**

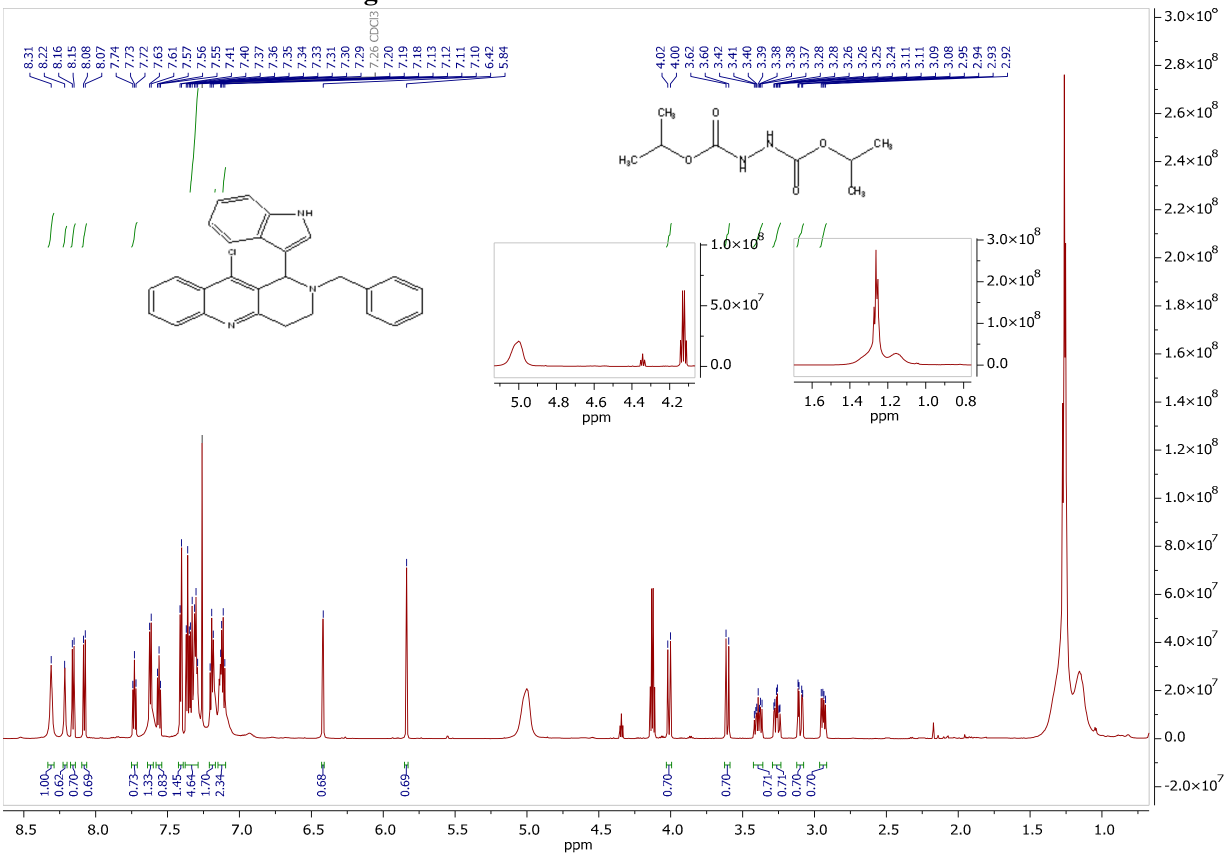

Figure S43. The 1H NMR data of **8c**

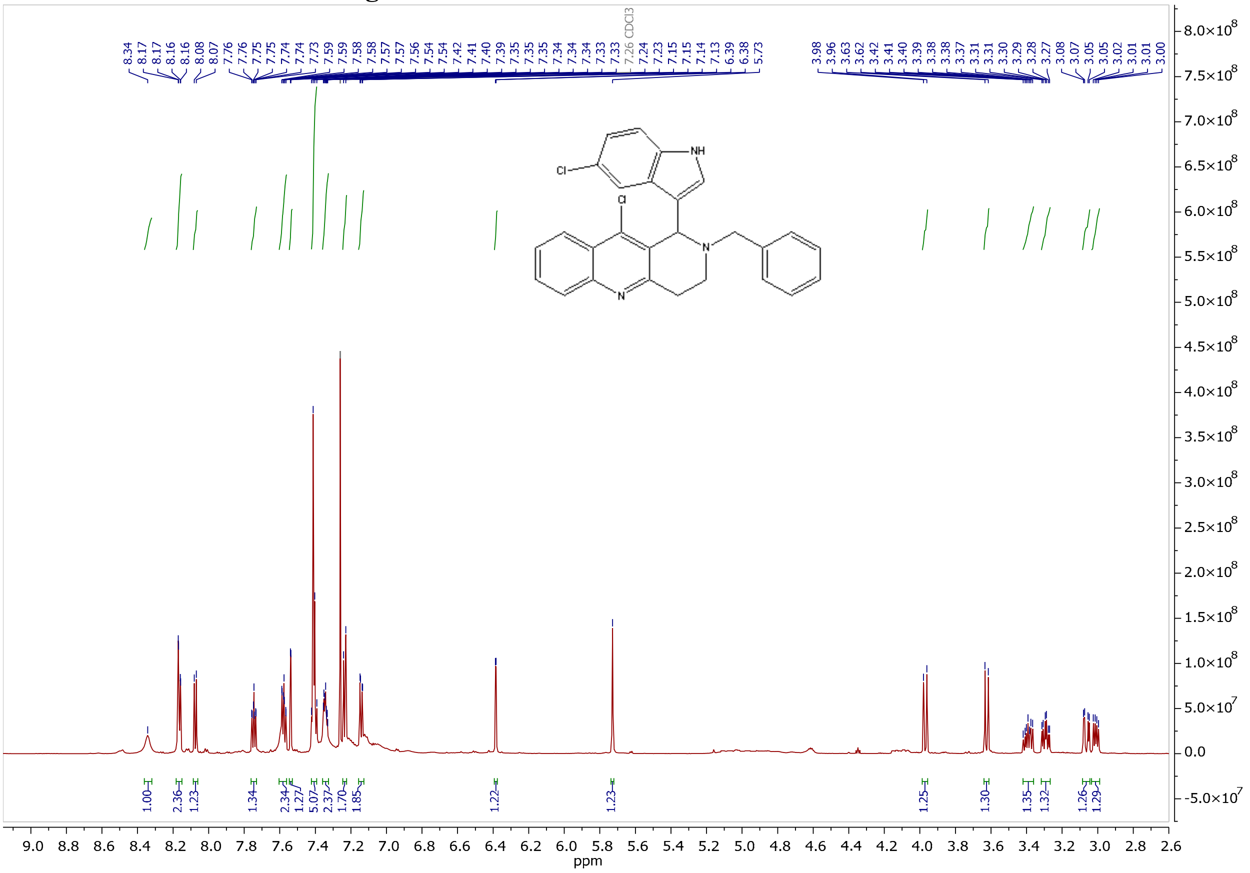

Figure S44. The 1H NMR data of **8d**

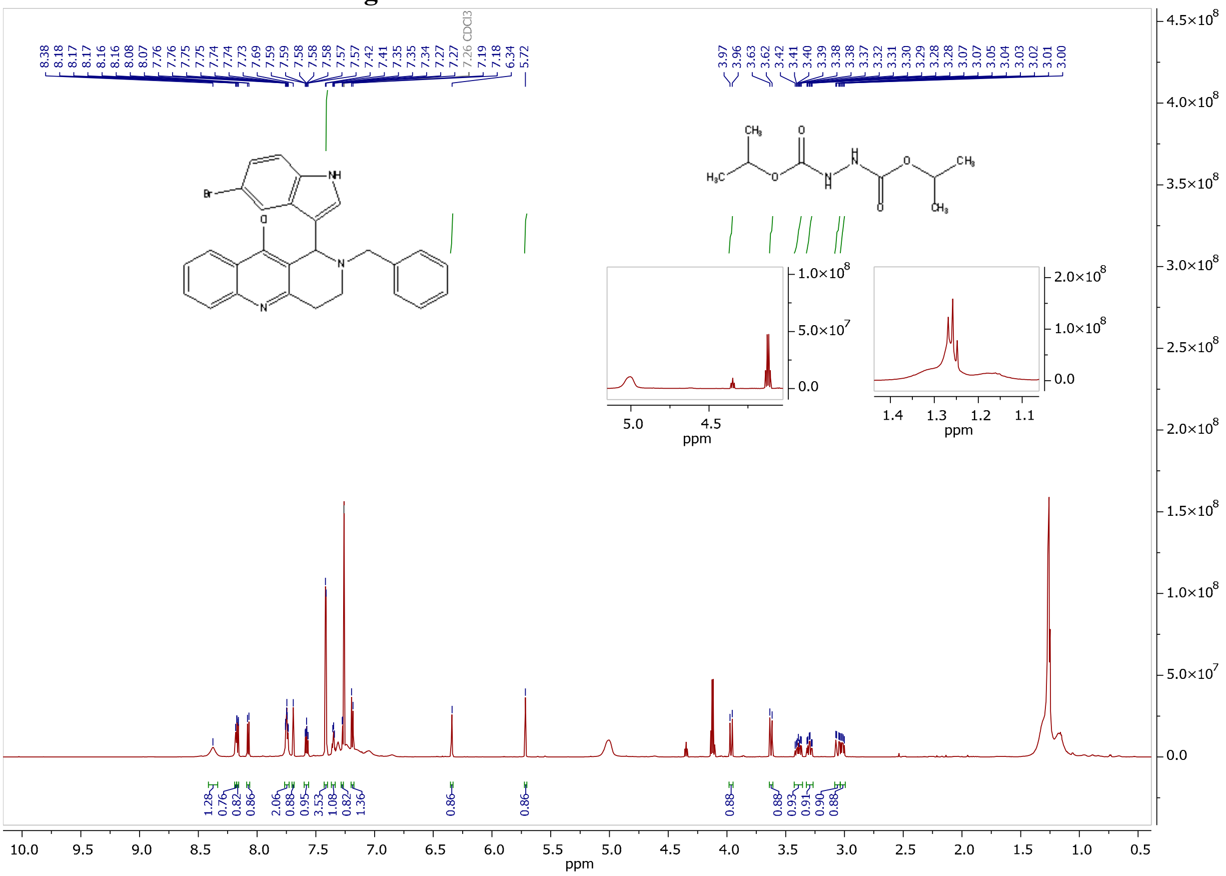

Figure S45. The 1H NMR data of **10a**

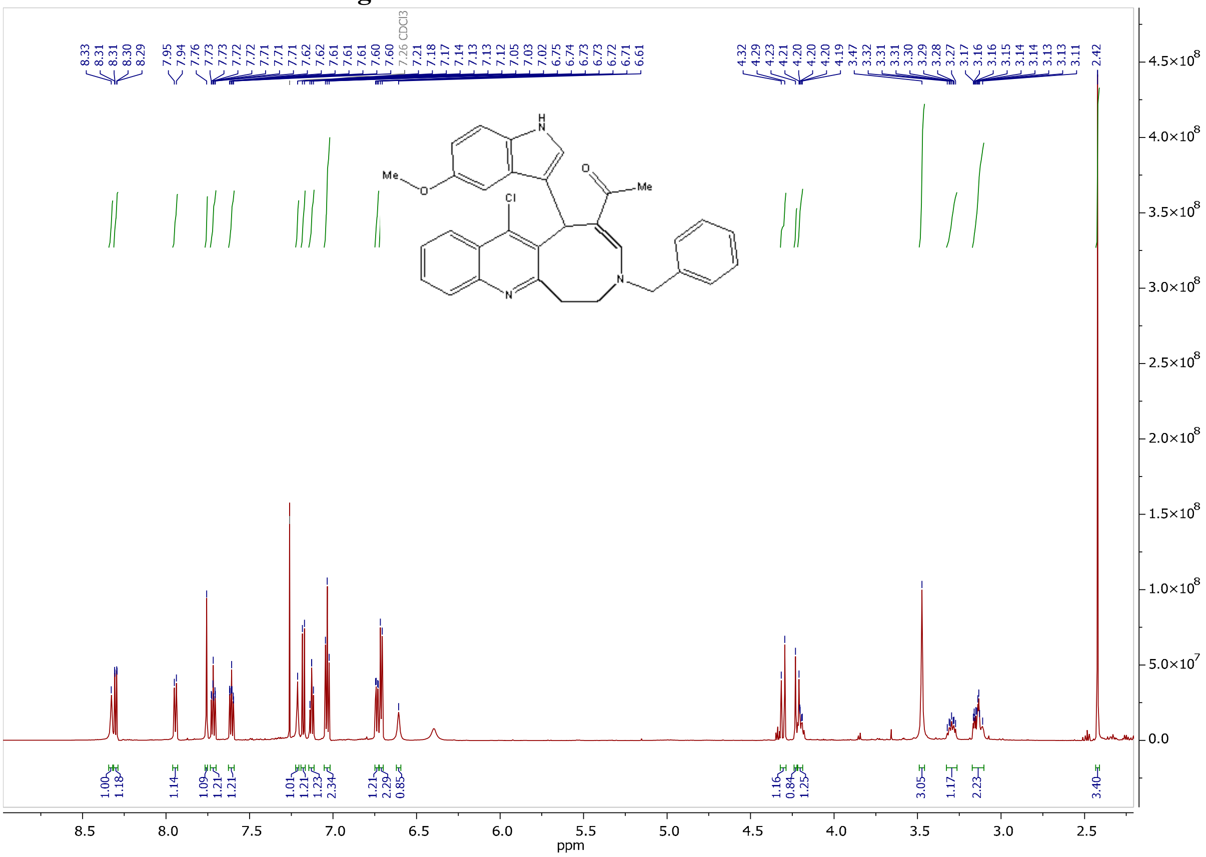

Figure S46. The 13C NMR data of **10a**

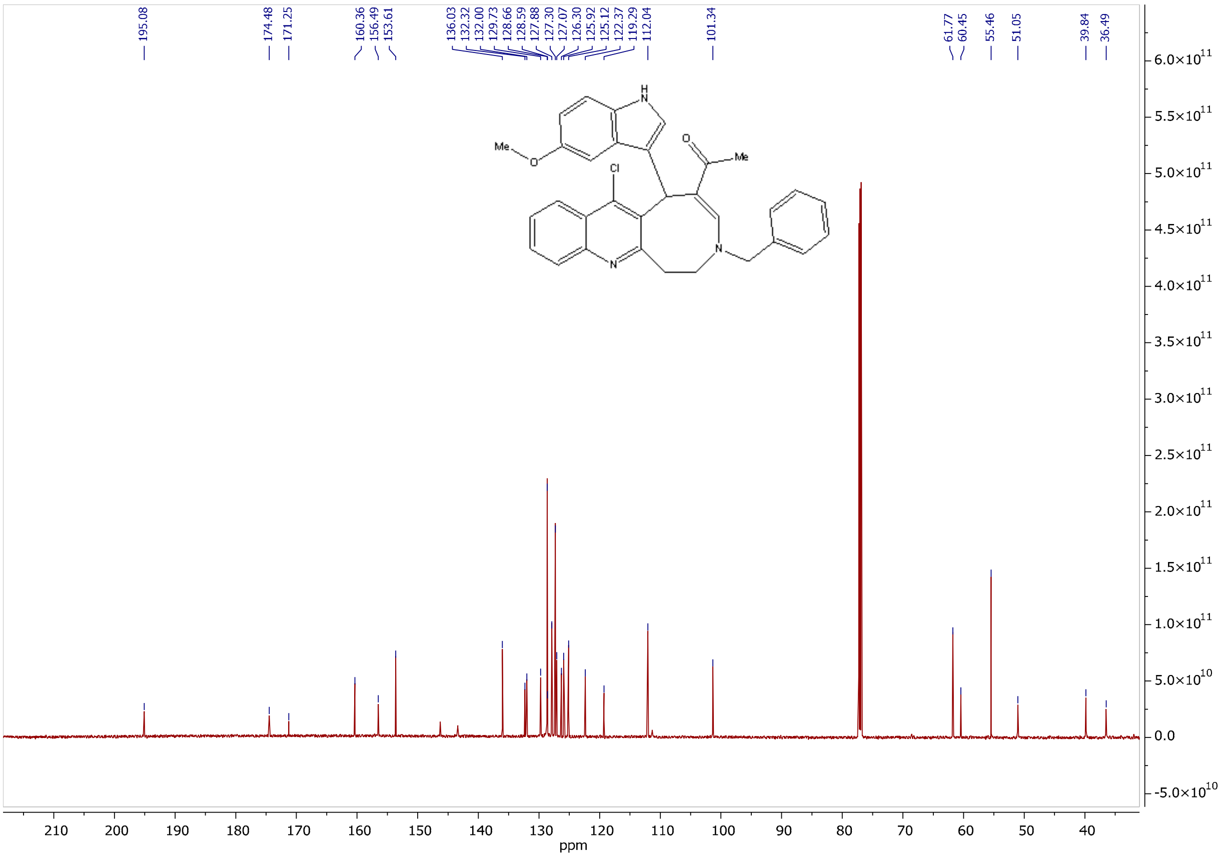

Figure S47. The 1H NMR data of **10b**

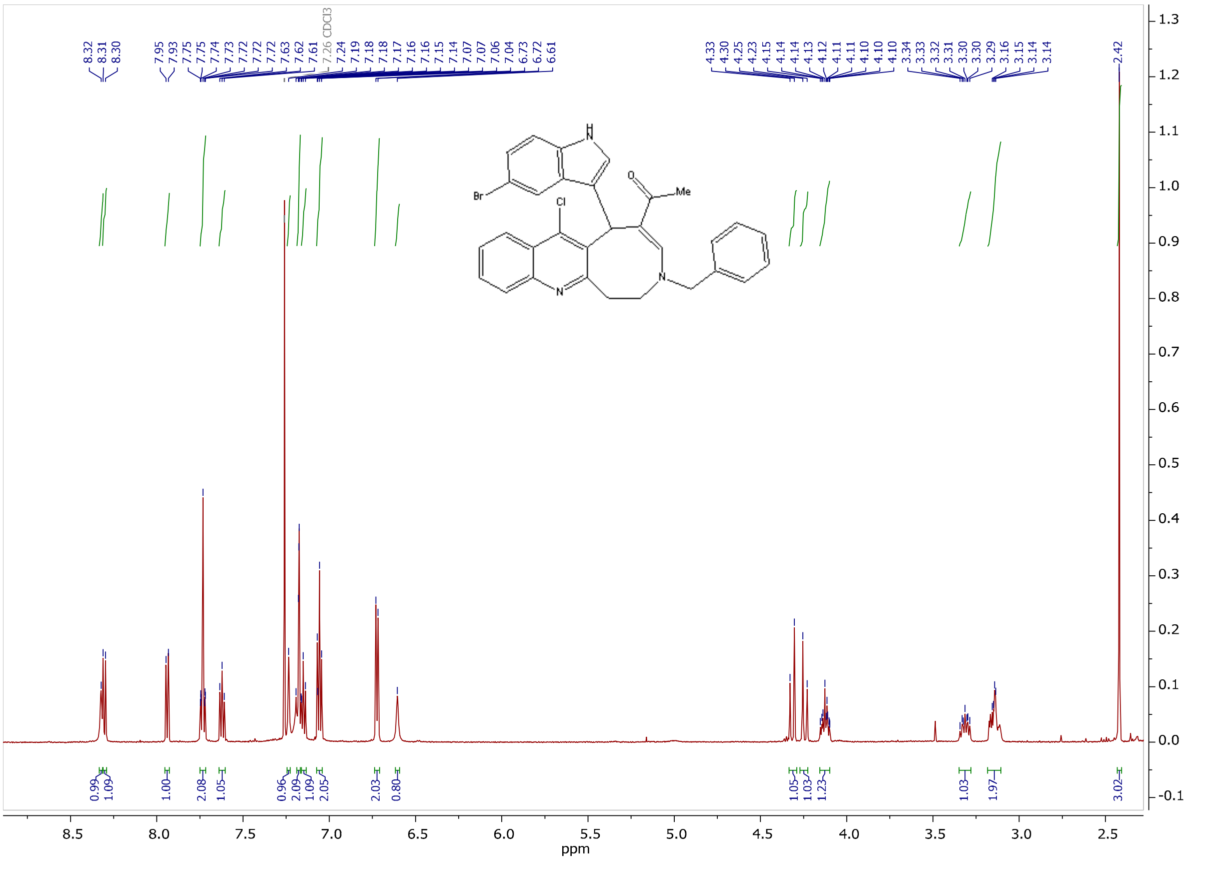

Figure S48. The 13C NMR data of **10b**

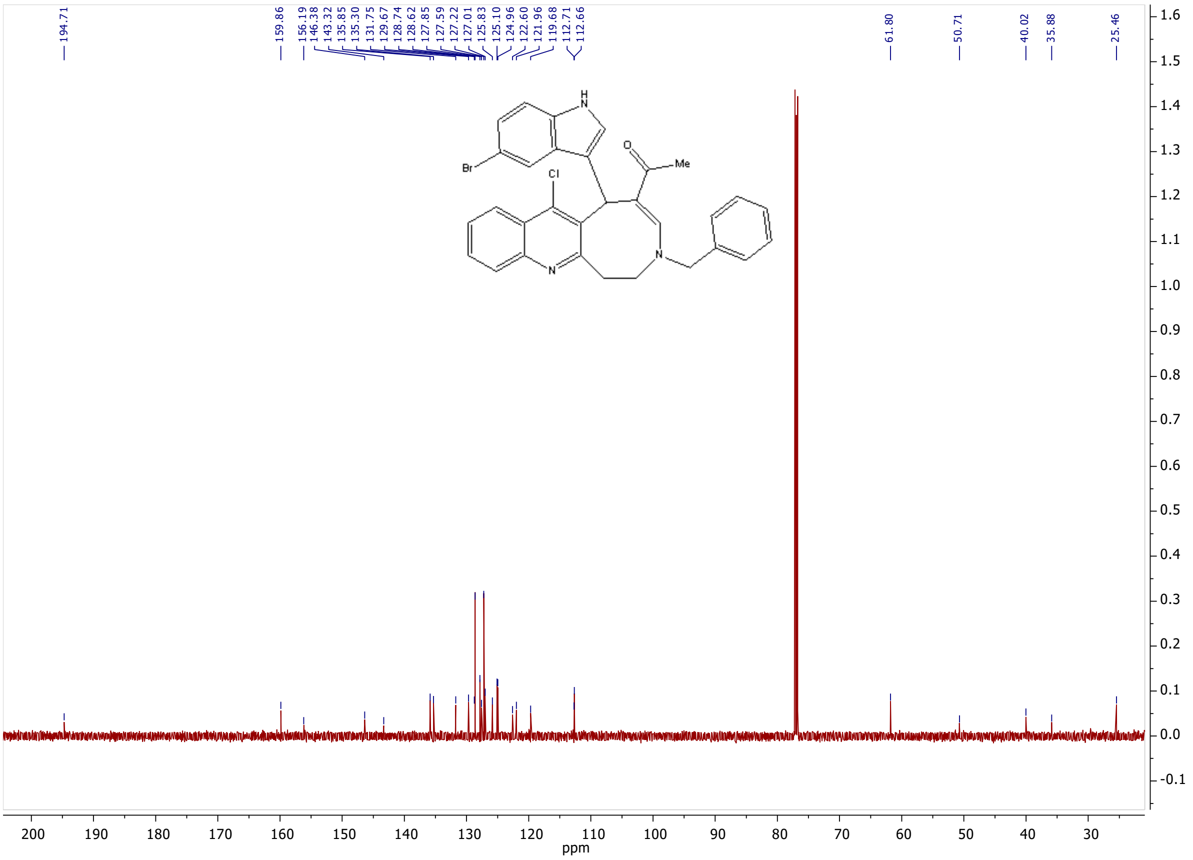

The crystallographic data compound **5a.**

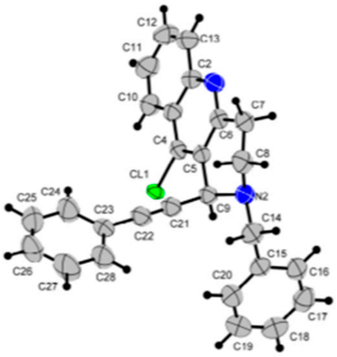

A single crystal X-ray analysis of compound **5a**.

Table S1. Crystal data and structure refinement for **5a**.

Empirical formula	C_{27} H_{21} Cl N_2
Formula weight	408.91
Temperature	295(2) K
Wavelength	1.54186 Å
Crystal system	Triclinic
Space group	P -1

Unit cell dimensions	a = 8.711(3) Å	α= 84.483(3)°.
	b = 9.2683(3) Å	β= 77.132(3)°.
	c = 14.6665(5) Å	γ = 67.091(3)°.
Volume	1063.2(4) $Å^3$	
Z	2	
Density (calculated)	1.277 Mg/m^3	
Absorption coefficient	1.698 mm^{-1}	
F(000)	428	
Theta range for data collection	5.181 to 67.500°.	
Index ranges	-9<=h<=10, -7<=k<=11, -16<=l<=17	
Reflections collected	10522	
Independent reflections	3611 [R(int) = 0.0414]	
Completeness to theta = 67.500°	94.2 %	
Refinement method	Full-matrix least-squares on F^2	
Data / restraints / parameters	3611 / 0 / 276	
Goodness-of-fit on F^2	0.834	
Final R indices [I>2sigma(I)]	R1 = 0.0372, wR2 = 0.0798	
R indices (all data)	R1 = 0.0698, wR2 = 0.0871	
Extinction coefficient	0.0067(4)	
Largest diff. peak and hole	0.152 and -0.191 e. $Å^{-3}$	
